# Current Innovations in Meat Fermentation with a View to Producing More Sustainable, Healthier and Safer Products

**DOI:** 10.3390/foods15173057

**Published:** 2026-08-28

**Authors:** Ciarán H. Crowley, Geraldine Duffy, Artur Gluchowski, Joe P. Kerry

**Affiliations:** 1 School of Food and Nutritional Sciences, University College Cork, T12 E138 Cork, Ireland; 116402292@umail.ucc.ie; 2 Food Safety Department, Teagasc Food Research Centre, Ashtown, D15 KN3K Dublin, Ireland; geraldine.duffy@teagasc.ie; 3 Food Gastronomy and Food Hygiene Department, Institute of Human Nutrition Sciences, Warsaw University of Life Sciences (WULS), 02-776 Warsaw, Poland; artur_gluchowski@sggw.edu.pl

**Keywords:** dry-fermented meats, food preservation, microbiology, food formulation, fermentation

## Abstract

Fermentation is a longstanding preservation process used in meat manufacture to improve product stability, safety and sensory quality. Renewed interest in fermented meats has stimulated research into reformulation and processing strategies intended to address concerns associated with salt, nitrate, nitrite, smoke-derived contaminants and animal fat while maintaining effective fermentation and product quality. This narrative review critically examines current innovations in fermented meat manufacture, including the replacement or reduction of conventional preservatives, smoking alternatives, fat reformulation, functional starter cultures, probiotics, prebiotics and bacteriocins. Developments in non-thermal and accelerated processing, edible and active packaging, intelligent monitoring systems and emerging culture-development technologies are also considered. Evidence indicates that selected functional cultures and formulation strategies can support preservative reduction, microbial control, curing, oxidative stability and sensory development, although their effectiveness is strain-, product- and process-dependent. Several processing and packaging technologies offer additional possibilities for improving manufacturing efficiency, shelf-life management and product safety, but their validation in fermented meat systems varies considerably. Potential environmental benefits, including reduced refrigeration, shorter processing or extended shelf life, cannot be assumed to confer lower overall environmental impacts and require comparative assessment of energy use, material inputs, food-waste effects and life-cycle performance. Future development should therefore prioritise product-specific validation, integrated hurdle-system assessment, regulatory compliance, consumer acceptance and comparative environmental evaluation.

## 1. Introduction

The global population increased from approximately 2.5 billion in 1950 to 8.0 billion in 2022 [1]. This growth, together with pressures on land, water, energy and other resources, has intensified the need to improve the efficiency and resilience of food production systems [2]. Approximately 1.3 billion tonnes of food intended for human consumption have historically been estimated to be lost or wasted annually, corresponding to approximately one-third of global food production [3]. Food preservation and shelf-life extension may contribute to addressing these challenges by limiting avoidable losses, although their overall environmental value depends on the resources, energy and materials required to achieve them. Fermentation is a longstanding means of food preservation. The International Scientific Association for Probiotics and Prebiotics defined fermented foods as “foods made through desired microbial growth and enzymatic conversions of food components” [4]. Archaeological evidence indicates the production of fermented beverages from rice, honey and fruit in Neolithic China, while meat drying and salting were practised in ancient societies and subsequently contributed to the development of fermented sausage manufacture [5]. Although the fundamental principles of fermentation, salting, curing and smoking remain recognisable, their application has evolved through improved control of raw materials, microbial cultures, processing conditions, packaging and product safety [6].

Interest in these traditional preservation processes has increased as manufacturers and researchers seek strategies that can support food safety, shelf life, product quality and more resource-efficient production [7]. Fermented meat manufacture can generate microbiologically stable products through the combined effects of acidification, reduced water activity, competitive microbiota, curing agents, smoking and drying [8]. Depending on product type and processing conditions, this may reduce reliance on certain thermal treatments or continuous refrigerated or frozen storage. These characteristics suggest potential sustainability advantages, but environmental performance cannot be inferred from the traditional or minimally processed character of a technology alone [9,10]. Comparative evidence concerning energy consumption, material requirements, product losses, distribution, storage and life-cycle impacts is required to determine whether a particular intervention provides an overall environmental benefit [11]. The composition and processing of fermented meats have also received scrutiny because of concerns associated with sodium, nitrate and nitrite, smoke-derived contaminants, animal fat composition and the health implications of processed-meat consumption. These concerns have encouraged research into reducing or replacing selected ingredients while maintaining microbiological safety, technological functionality, sensory quality and shelf life [12,13]. Current approaches include the use of alternative curing systems, salt and fat replacers, controlled smoking technologies, functional starter cultures, protective microorganisms, bacteriocins and combinations of biological and physical hurdles. Emerging processing and packaging technologies may provide additional means of controlling microorganisms, accelerating manufacture, protecting product quality and monitoring conditions during distribution [14]. However, their evidential maturity and suitability for fermented meat applications vary considerably.

This review critically examines current innovations in fermented meat formulation, fermentation microbiology, processing and packaging with a view to supporting the production of safer, healthier and potentially more sustainable products. It considers strategies for reducing or replacing nitrate, nitrite, salt, smoke and animal fat; developments in starter cultures, probiotics, prebiotics and bacteriocins; and emerging biological, non-thermal, accelerated processing and packaging technologies. Attention is given to the strength and directness of the available evidence; the distinction between laboratory potential and validated product-level performance; and the technological, sensory, regulatory and environmental considerations affecting commercial implementation. The literature for this narrative review was identified through an iterative search conducted over approximately five years and updated throughout manuscript development, with the search most recently updated in August 2026. Principal searches were undertaken using Web of Science, PubMed, ScienceDirect, SpringerLink and Google Scholar and were supplemented by searches of relevant specialist journals and online research-discovery platforms. Search terms initially focused on dry-fermented meats and were subsequently broadened according to the topic under consideration. Terms relating to fermented meat, dry-fermented sausage, nitrate and nitrite reduction, salt reduction, smoking, fat replacement, starter and functional cultures, probiotics, prebiotics, bacteriocins, non-thermal and accelerated processing, edible and active packaging, intelligent packaging, food safety, consumer acceptance and sustainability were used individually and in combination. Sources were selected according to their relevance to the scope of the review, the directness of the evidence provided and their contribution to established or emerging developments in fermented meat manufacture. Although no formal restrictions were imposed concerning publication date, language or publication type, preference was given to peer-reviewed articles published in established journals. Official legislative and institutional sources, including European Union legislation and publications from relevant food-safety authorities, were used for regulatory and risk-assessment information. Evidence from adjacent food systems was included selectively where direct fermented meat evidence was limited and the underlying technological principle was relevant; such evidence was treated as indicating a possible future application and not as direct validation in fermented meat products.

## 2. Innovations in the Replacement of Chemical Preservatives in Fermented Meat Products

### 2.1. Nitrates and Nitrites

#### 2.1.1. The Technological Importance of Nitrate and Nitrite

Nitrate and nitrite have traditionally been used in fermented meat manufacture because they contribute to several interrelated aspects of product safety and quality. Nitrate functions principally as a reservoir that must first be reduced to nitrite by nitrate-reducing microorganisms. Under the reducing and acidic conditions that develop during fermentation, nitrite subsequently generates nitric oxide and other reactive nitrogen species. Nitric oxide binds to the haem iron of myoglobin to form nitrosylmyoglobin, which is converted during heating or maturation into the characteristic stable pigments associated with cured-meat colour. Nitrite also delays lipid oxidation by interacting with haem proteins, chelating pro-oxidant metal ions and limiting iron-mediated radical formation, thereby contributing to flavour stability and extending product shelf life [15,16,17]. The antimicrobial activity of nitrite is particularly important for controlling *Clostridium botulinum*, although its efficacy depends on the amount of nitrite added and retained, product pH, salt concentration, water activity, temperature and the presence of other antimicrobial hurdles. Rather than acting through a single mechanism, nitrite-derived reactive nitrogen species interfere with several microbial targets, including iron–sulphur enzymes involved in cellular energy metabolism. This disruption can inhibit vegetative-cell growth and spore outgrowth, thereby reducing the risk of toxin production. Nitrite may also contribute to the control of other pathogenic and spoilage microorganisms; however, its antimicrobial action should be regarded as one component of the wider hurdle system used in fermented meat manufacture rather than as an independent guarantee of product safety [15,18,19].

#### 2.1.2. The Physiological Metabolism of Nitrate and Nitrite

Nitrate and nitrite are also involved in mammalian physiology through the nitrate–nitrite–nitric oxide pathway. Nitrate originates both from dietary exposure principally through vegetables and drinking water and from the endogenous oxidation of nitric oxide. Following intestinal absorption, a proportion of circulating nitrate is actively taken up and concentrated by the salivary glands before being secreted into saliva. The glands therefore concentrate and recirculate nitrate rather than synthesising it [16]. Commensal bacteria on the tongue reduce salivary nitrate to nitrite, which is swallowed and may subsequently be converted into nitric oxide and other bioactive nitrogen species in the acidic gastric environment and within tissues. Nitric oxide is an important signalling molecule involved in vascular tone, platelet activity and other physiological processes [20]. Accordingly, controlled dietary nitrate interventions (particularly those involving nitrate-rich vegetables) have been associated with reductions in blood pressure and changes in vascular function in some populations [21,22,23,24]. Nevertheless, nitrate is not currently classified as an essential dietary nutrient, and reported physiological benefits should be distinguished from the toxicological and epidemiological questions associated with nitrate and nitrite exposure from different dietary sources.

The physiological roles of the nitrate–nitrite–nitric oxide pathway do not preclude adverse effects under different chemical and dietary conditions. Nitrite can react with secondary amines and amides to form *N*-nitroso compounds (NOCs), a diverse group that includes *N*-nitrosamines and *N*-nitrosamides, several of which have demonstrated genotoxic and carcinogenic properties. These reactions may occur during food processing, storage or high-temperature cooking, as well as endogenously following ingestion. Nitrosation is favoured by acidic conditions and depends on the availability of nitrosating species and suitable precursors; consequently, dietary nitrate or nitrite exposure cannot be interpreted independently of the chemical environment in which it occurs. Other toxicological effects also remain relevant: excessive nitrite exposure can oxidise haemoglobin to methaemoglobin and impair oxygen transport, an effect that has informed the establishment of acceptable daily intakes for nitrite and nitrate [16,17].

#### 2.1.3. Toxicology, Nitrosation and Carcinogenicity

The food matrix is therefore central to understanding the health implications of nitrate and nitrite. Vegetables constitute a major source of dietary nitrate but also provide vitamin C, polyphenols and other antioxidant compounds capable of limiting nitrosative reactions. Their consumption is consequently not considered biologically equivalent to exposure through processed meat solely because both food categories may contain nitrate. In processed meats, nitrite-derived species may interact with amines, amides, lipids and haem-containing proteins during manufacture, digestion and cooking. Haem iron can promote lipid peroxidation and endogenous formation of apparent total *N*-nitroso compounds in the gastrointestinal tract, while the resulting products of nitrosation and oxidation may contribute to cytotoxicity and genotoxicity in the colonic environment. Conversely, the inclusion of ascorbate or erythorbate in cured meat formulations can inhibit some nitrosation reactions. The balance between promoting and inhibiting factors is affected by formulation, residual nitrite concentration, processing conditions, storage, cooking method and the wider composition of the meal. Nitrate concentration alone is therefore an inadequate measure of the potential health effect of a food [17,25,26,27].

Epidemiological findings concerning nitrate and nitrite must similarly be interpreted according to exposure source and cancer site. Studies of dietary nitrate, drinking-water nitrate, nitrate-rich vegetables and processed-meat consumption examine related but non-equivalent exposures and have not produced uniform results [25,28,29,30,31]. Some systematic reviews have reported associations between nitrate or nitrite exposures and selected cancers, whereas others have found weak, inconsistent or non-significant relationships. Interpretation is complicated by differences in exposure assessment, dietary patterns, water sources, follow-up periods and adjustment for potential confounders. Evidence concerning nitrate-rich vegetables should not be used either to establish the safety of nitrite-containing processed meats or to infer that nitrate itself is uniformly harmful. The available evidence instead indicates that nitrate and nitrite participate in both beneficial physiological pathways and potentially harmful reactions, with the net effect determined substantially by dose, source, food matrix and conditions of exposure.

#### 2.1.4. Institutional Standpoint on Nitrate and Nitrite

At the level of the whole food, the International Agency for Research on Cancer classified the consumption of processed meat as carcinogenic to humans (Group 1), based on sufficient evidence that processed-meat consumption causes colorectal cancer. This classification concerns processed meat as a dietary exposure and does not identify added nitrate or nitrite as its sole causal component. The potential mechanisms considered include the formation of NOCs, oxidative reactions involving ham-iron and compounds generated during smoking or high-temperature cooking. Moreover, a Group 1 classification describes the strength of the evidence that an exposure can cause cancer; it does not imply that processed meat presents the same magnitude of risk as other agents within that category. The IARC estimated that each 50 g portion of processed meat consumed daily was associated with an approximately 18% increase in the relative risk of colorectal cancer while noting that individual risk is influenced by the amount and frequency of consumption [32,33]. The European Food Safety Authority has addressed a related but distinct question through its risk assessments of nitrate and nitrite as food additives. In its 2017 re-evaluations, EFSA established an acceptable daily intake of 0.07 mg nitrite/kg body weight per day and retained an acceptable daily intake of 3.7 mg nitrate/kg body weight per day. EFSA concluded that exposure arising specifically from authorised food-additive uses was generally within these health-based guidance values, although total exposure from all dietary sources could exceed the relevant acceptable daily intake in some population groups. EFSA also recognised uncertainty surrounding the formation and dietary occurrence of NOCs [34,35]. Its subsequent assessment concluded that dietary exposure to certain *N*-nitrosamines in food raises a health concern while identifying substantial uncertainties and data gaps concerning their occurrence and formation. These conclusions are not contradictory to the IARC’s classification: the IARC evaluated whether consumption of processed meat can cause cancer, whereas EFSA assessed defined chemical exposures against health-based guidance values [36]. Together, these evaluations support continued efforts to reduce unnecessary nitrate and nitrite exposure and NOC formation without disregarding the important microbiological and technological functions of curing salts.

The available evidence indicates that the relationship between nitrate, nitrite and human health cannot be characterised as uniformly beneficial or harmful. Their biological effects depend on dose, chemical transformation, exposure source, food-matrix composition and the conditions under which products are manufactured, stored, prepared and consumed. In fermented meats, nitrate and nitrite provide interdependent antimicrobial, antioxidant, sensory and colour-forming functions that cannot necessarily be reproduced through the removal of a single ingredient. Nevertheless, concerns surrounding dietary exposure, endogenous nitrosation and the formation of potentially carcinogenic *N*-nitroso compounds provide a clear rationale for reducing their use where technological safety can be maintained. Innovation in this area should therefore be understood as a process of risk–benefit optimisation rather than the indiscriminate elimination of curing salts. Current approaches include reducing directly added nitrate and nitrite; employing plant-derived nitrate in combination with nitrate-reducing cultures; incorporating antioxidant or antimicrobial ingredients to compensate for selected curing functions; promoting alternative pigment-forming pathways such as zinc protoporphyrin IX formation; and using microbial nitric oxide-generating systems. These strategies differ substantially in mechanism and do not necessarily eliminate nitrate-, nitrite- or nitric oxide-mediated chemistry. Their suitability must consequently be evaluated according to microbiological safety, residual curing-agent concentrations, oxidative stability, colour and flavour development, sensory acceptance and regulatory compliance. Against this background, the following sections evaluate each reformulation strategy according to its underlying mechanism, the curing functions it seeks to retain or replace, and the strength of evidence supporting its application in fermented meat systems. Direct reduction is considered first because it preserves conventional nitrate- and nitrite-mediated curing chemistry while attempting to reduce the degree of reliance upon it.

#### 2.1.5. Nitrite Reduction Strategies in Dry-Fermented Meats

- Direct reduction within a multi-hurdle system

The most direct approach to limiting dietary exposure is to reduce the concentration of conventionally added nitrate and nitrite while retaining sufficient curing activity to support product safety and quality. In fermented meats, such reductions may be facilitated through hurdle technology, whereby several individually sublethal preservation factors act collectively to restrict microbial survival and growth. Rapid acidification by lactic acid bacteria, progressive reduction of water activity during drying, salt, competitive or antimicrobial starter cultures, low processing and storage temperatures, thermal treatment and protective packaging may therefore compensate, to varying degrees, for reduced reliance on curing salts. These factors are interdependent: acidification can increase the antimicrobial efficacy of nitrite, while lower pH and water activity progressively restrict the growth of pathogenic and spoilage microorganisms during fermentation and maturation. However, the contribution of each hurdle depends on the product formulation, manufacturing process and target organism, and reducing nitrate or nitrite may also affect cured-colour formation, oxidative stability and sensory quality. Consequently, no universally applicable minimum concentration can be inferred from successful reduction in a single product. Reduced-nitrite formulations must instead be validated within the complete product system using physicochemical measurements, microbial challenge studies where appropriate and assessment throughout the intended shelf life [37,38,39,40,41].

- Indirect curing using plant-derived nitrate

Indirect curing employs nitrate-rich plant ingredients, such as celery, radish, beetroot or leafy-vegetable powders and extracts, as alternatives to purified nitrate or nitrite salts. In these systems, nitrate supplied by the plant material is reduced to nitrite by nitrate-reducing microorganisms, commonly through the addition of a starter culture containing coagulase-negative staphylococci such as *Staphylococcus carnosus*. The resulting nitrite may subsequently generate nitric oxide and contribute to cured-colour development, oxidative stability, flavour and microbial inhibition through broadly the same chemical pathways associated with conventionally added curing salts. Plant-derived nitrate should therefore be regarded as an alternative source of curing agents rather than as their complete replacement [42]. Although this approach may support clean-label reformulation and respond to consumer preferences for recognisable ingredients, it does not necessarily eliminate residual nitrate or nitrite, nitric oxide-mediated curing chemistry or the potential formation of *N*-nitroso compounds. Its technological effectiveness is also influenced by the nitrate concentration and composition of the plant ingredient, the activity of the nitrate-reducing culture, fermentation temperature and duration, product pH and the time available for conversion. Variability in these factors may produce inconsistent nitrite formation and curing performance; consequently, plant-derived curing systems require standardisation and product-specific validation of residual curing-agent concentrations, colour, oxidative stability and microbiological safety [15,17,43,44,45].

- Functional compensation using antioxidant and antimicrobial ingredients

Plant extracts, essential oils and other naturally derived bioactive ingredients may facilitate nitrate or nitrite reduction by compensating for selected functions of curing salts rather than by supplying nitrate or generating nitrite. Polyphenols, flavonoids, phenolic acids and related phytochemicals can inhibit lipid and protein oxidation through radical scavenging, metal chelation and interactions with pro-oxidant haem components. Certain plant-derived compounds may additionally inhibit pathogenic or spoilage microorganisms through disruption of cellular membranes, interference with enzyme activity or modification of microbial energy metabolism. Ingredients investigated in fermented meats include grape seed and chestnut extracts, hydroxytyrosol, *Kitaibelia vitifolia* extract, mulberry polyphenols, olive-derived phenolics and essential oils. Their effectiveness is influenced by botanical source, extraction method, phytochemical composition, concentration, interactions with the meat matrix and stability during fermentation, drying and storage. Moreover, concentrations sufficient to produce antioxidant or antimicrobial effects may also alter flavour, aroma, colour or fermentation microbiota. These ingredients should therefore be described as partial functional substitutes or compensatory adjuncts because control of lipid oxidation or selected microorganisms does not establish equivalent cured-colour formation, flavour development or protection against *Clostridium botulinum*. Their application is most defensible as part of a validated multi-hurdle system in which the function replaced and the curing functions that remain uncompensated are explicitly identified [46,47,48,49,50,51].

- Alternative colour formation through zinc protoporphyrin IX

Zinc protoporphyrin IX (ZnPP) formation represents an alternative strategy for developing stable red colour in nitrate- and nitrite-free meat products. ZnPP is the pigment principally associated with the characteristic red colour of traditionally manufactured Parma ham, in which nitrate and nitrite are not added. Its formation involves the replacement of iron in the haem structure by zinc, producing a red, fluorescent and comparatively stable metalloporphyrin [52]. Current evidence indicates that ZnPP may arise through both enzymatic and non-enzymatic pathways, including ferrochelatase-mediated incorporation of zinc into protoporphyrin IX and transmetallation involving the displacement of iron from haem by zinc. The relative contribution of these mechanisms appears to depend on the meat matrix and processing environment. ZnPP formation is influenced by meat species and muscle composition, pH, temperature, oxygen availability, zinc and iron availability, microbial activity and maturation time. Nitrite can strongly inhibit its formation, partly because nitrite-derived nitric oxide binds to myoglobin and diverts the pigment chemistry towards nitrosylated haem [53,54]. Consequently, ZnPP formation is most relevant to products manufactured without nitrite or with very low residual nitrite concentrations.

Although first characterised principally in long-ripened whole-muscle products, ZnPP has also been detected in nitrite-free dry-fermented sausages. Its formation in these products may be promoted through the control of raw-material and processing conditions or through selected ZnPP-forming microorganisms. Food-grade lactic acid bacteria, including strains of *Lactococcus lactis* and *Latilactobacillus curvatus*, have demonstrated the capacity to promote ZnPP formation and improve redness in experimental nitrite-free dry-cured sausages [55]. More recent screening studies have identified considerable strain-dependent variation among lactic acid bacteria, indicating that ZnPP production is not a general characteristic that can be assumed from species identity alone. Nevertheless, fermented sausages present additional challenges compared with slowly matured whole-muscle products because acidification, shortened production time, microbial interactions and the physical distribution of fat and lean tissue may influence pigment formation. Starter-assisted ZnPP production should therefore be regarded as an emerging technology requiring strain-level selection and validation under the intended manufacturing conditions [56,57]. Importantly, ZnPP provides colour compensation rather than complete nitrite replacement. The formation of a stable red pigment does not itself reproduce nitrite-mediated inhibition of *Clostridium botulinum*, the control of lipid oxidation or the development of conventional cured flavour [19]. A ZnPP-forming system must therefore be combined with other validated hurdles where nitrate and nitrite are removed. Assessment should extend beyond instrumental redness to include pigment identity and stability, oxidative changes, pathogenic and spoilage microorganisms, fermentation performance, sensory acceptance and shelf life [58]. Accordingly, ZnPP is best understood as one component of a broader nitrate- and nitrite-free preservation system rather than as an independent substitute for curing salts.

- Nitric oxide-mediated nitrosylation through bacterial nitric oxide synthase

Bacterial nitric oxide synthase (bNOS) provides a second emerging route to cured-like colour formation in products manufactured without directly added nitrate or nitrite. Certain meat-associated bacteria, particularly selected coagulase-negative staphylococci, possess nitric oxide synthase activity capable of converting L-arginine to nitric oxide and L-citrulline in an oxygen-dependent reaction [59]. The nitric oxide produced may bind to the haem iron of myoglobin, forming nitrosylmyoglobin and thereby promoting the characteristic red colour associated with cured meat. This pathway differs from conventional and plant-derived curing because nitrate reduction is not required as the immediate source of nitric oxide: (L-arginine → bacterial nitric oxide synthase → nitric oxide → nitrosylmyoglobin). However, the final pigment-forming reaction remains based on nitric oxide-mediated nitrosylation [60]. The strategy may therefore avoid the direct addition or microbial reduction of nitrate and nitrite, but it does not replace nitrosylation chemistry with an entirely different colour-forming mechanism. The technological expression of this pathway is strongly strain- and process-dependent [61]. Genes encoding nitric oxide synthase have been identified in several meat-associated staphylococci, and studies using *Staphylococcus xylosus* have confirmed NOS-dependent nitric oxide production and the formation of red myoglobin derivatives [62,63]. Nevertheless, possession of a *nos* gene does not necessarily predict sufficient phenotypic activity under meat-fermentation conditions. L-arginine may also be metabolised through competing pathways, particularly the arginine deiminase and arginase systems, while nitric oxide synthesis depends on oxygen availability, suitable electron-donor systems, substrate concentration and environmental conditions [64]. Product pH, temperature, redox potential, bacterial competitiveness and oxygen gradients within the sausage may consequently influence nitric oxide production and pigment uniformity. These limitations are particularly relevant in large-calibre fermented sausages, where progressively restricted oxygen availability towards the centre may constrain the oxygen-dependent NOS reaction. Screening and selection must therefore be undertaken at the strain level and confirmed in the intended meat matrix rather than inferred from species identity or gene detection alone [65,66].

Early meat-model research illustrated these constraints: a nitric oxide synthase-positive *Staphylococcus haemolyticus* strain failed to establish satisfactory colour after being outcompeted by the background microbiota, raising doubts about the practical robustness of the pathway under conventional fermentation conditions [67]. More recent studies, however, provide stronger evidence of technological potential. NOS activity has been experimentally linked to nitric oxide and red-pigment formation in *S. xylosus*, while investigations of multiple coagulase-negative staphylococcal strains have demonstrated substantial variation in NOS activity and interactions with other routes of arginine metabolism [68]. In *Staphylococcus vitulinus* (now classified as *Mammaliicoccus vitulinus*), enhanced NOS expression increased nitric oxide production, nitrosylmyoglobin formation and redness in fermented sausage. Subsequent product-level research similarly reported improved redness and nitrosylmyoglobin formation following inoculation with *M. vitulinus* [69,70]. Collectively, these findings indicate that bacterial NOS-mediated colour formation is biologically plausible and increasingly supported experimentally but remains an emerging and strain-dependent technology rather than an established universal curing system.

As with ZnPP, bacterial nitric oxide generation should currently be regarded primarily as colour compensation rather than complete nitrate or nitrite replacement. Nitric oxide-mediated nitrosylmyoglobin formation may reproduce cured redness, but it does not by itself demonstrate equivalent inhibition of *Clostridium botulinum*, oxidative stability, cured-flavour development or shelf-life protection [17]. Moreover, generating nitric oxide from L-arginine does not establish that the resulting product is free from all nitrosative chemistry. The fate and concentration of the nitric oxide produced via its reactions with meat components and its potential contribution to nitrosated compounds require investigation under realistic processing and gastrointestinal conditions. Any bNOS-based system must therefore be evaluated within a broader hurdle strategy, including assessment of pathogen control, oxidation, pigment identity and distribution, sensory quality, starter-culture safety and stability throughout shelf life.

- Deliberately uncured product reformulation

A further strategy is the deliberate development of uncured fermented meats in which nitrate and nitrite are neither directly added nor introduced through nitrate-rich or pre-converted plant ingredients. This approach does not necessarily seek to preserve nitric oxide-dependent curing chemistry or reproduce every characteristic of a conventionally cured product. Instead, microbiological stability may be established through an alternative combination of rapid fermentation, salt, progressive reduction of water activity, protective cultures, antimicrobial metabolites, thermal treatment, hygienic manufacture, suitable packaging and controlled storage [71]. Product colour, flavour, aroma and oxidative behaviour may consequently differ from those of conventional fermented meats. Such differences should not automatically be interpreted as technological defects; they may form part of the sensory identity of a genuinely distinct uncured product, provided that the formulation remains acceptable to consumers and demonstrably safe throughout its intended shelf life [72]. Complete removal of nitrate and nitrite nevertheless creates important technological challenges. Acidification and drying can strongly restrict many vegetative pathogens, but their effectiveness depends on the rate and extent of pH and water activity reduction, fermentation temperature, salt concentration, product dimensions and the resistance of the target organism [73]. Moreover, the absence of nitrite may reduce protection against spore-forming bacteria and remove an important safeguard against *Clostridium botulinum* growth and toxin production. It may also increase susceptibility to lipid and pigment oxidation, discolouration, rancid flavour development and reduced storage stability. Acceptable microbial counts, colour or oxidative stability at the completion of manufacture therefore cannot establish safety and quality over the full commercial life of the product [74]. Uncured formulations must be validated as complete systems under reasonably foreseeable processing, packaging, storage and temperature-abuse conditions, using physicochemical characterisation, relevant pathogen challenge testing, oxidation measurements, sensory evaluation and shelf-life assessment [75]. Deliberately uncured reformulation should therefore be understood as product redesign rather than one-for-one ingredient replacement. The objective is not to identify a single compound capable of assuming every function of nitrite but to redistribute those functions across formulation, microbial ecology, processing and storage. Rapid acidification and reduced water activity may provide microbial control; selected cultures or antimicrobial ingredients may reinforce pathogen inhibition; antioxidants and oxygen-restrictive packaging may limit oxidation; and consumer expectations may be adapted to a product with a different colour or flavour profile. The adequacy of these measures must, however, be demonstrated collectively rather than inferred from the performance of any individual hurdle. Where equivalent safety, stability and sensory acceptance can be established, the uncured product may represent a legitimate new category rather than an incomplete imitation of conventionally cured fermented meat.

Collectively, nitrate- and nitrite-reduction strategies range from modification of the conventional curing system to the development of fundamentally different fermented meat products. Direct reduction retains conventional curing salts at lower concentrations, whereas plant-derived curing changes their source without eliminating nitrate-, nitrite- or nitric oxide-mediated chemistry. Antioxidant and antimicrobial ingredients compensate for selected technological functions, while ZnPP formation and bacterial nitric oxide synthase provide distinct routes to cured-like colour. Deliberately uncured reformulation goes further by redesigning the preservation system without necessarily reproducing conventional curing chemistry or sensory characteristics. These approaches should not be evaluated as interchangeable substitutes. Their suitability depends on the functions they demonstrably perform and on whether microbial safety, oxidative stability, sensory quality and shelf life are maintained within the complete product system. Case studies of these reformulation strategies are presented in Table 1.

#### 2.1.6. The Regulation of Nitrate and Nitrite in the EU and USA

The regulatory treatment of nitrate- and nitrite-reduction strategies differs substantially between the European Union and the United States, particularly regarding permitted concentrations, ingredient classification and the meaning of “uncured” products. Within the European Union, potassium nitrite (E 249), sodium nitrite (E 250), sodium nitrate (E 251) and potassium nitrate (E 252) are regulated as food additives under Regulation (EC) No 1333/2008 [76]. Their use is product-specific, with separate provisions for non-heat-treated meat products and for named traditional or traditionally cured products. Commission Regulation (EU) 2023/2108 introduced lower limits and revised the basis on which concentrations are expressed, following reassessment of dietary exposure and the balance between controlling nitrosamine formation and retaining protection against pathogenic bacteria [77]. Since 9 October 2025, the general maximum amount of nitrite permitted to be added to meat products was reduced to 80 mg/kg, expressed as nitrite ion, while lower or product-specific limits apply to certain categories. Specific provisions remain for traditionally manufactured dry-fermented products; for example, *saucisson sec* and similar raw fermented sausages manufactured without added nitrite may contain up to 180 mg/kg nitrate, expressed as nitrate ion, subject to defined fermentation, maturation and water-to-protein criteria. Maximum residual concentrations may also apply [77]. These values must therefore be interpreted according to the relevant food category, whether they concern an ingoing or residual amount and whether the concentration is expressed as the ion or its sodium or potassium salt. Direct numerical comparison with older studies or non-EU legislation can otherwise be misleading. The EU framework does not establish a distinct “natural curing” category based solely on the botanical origin of nitrate or nitrite. The regulatory status of a vegetable powder, extract, microbial preparation or other alternative therefore depends on its composition, purpose and conditions of use rather than on a general distinction between natural and synthetic curing. Where a substance is used to perform a technological function in the finished product, questions may arise concerning its treatment as an additive rather than an ordinary food ingredient. Novel extracts, microorganisms or processes may additionally require assessment under applicable novel-food legislation, while starter cultures and other microbial interventions must satisfy general food-safety requirements and be suitable for their intended use. Consequently, replacing purified curing salts with nitrate-rich plant material does not necessarily remove nitrate exposure, nitrite formation or nitrosation chemistry, nor does botanical origin alone demonstrate regulatory acceptability or toxicological superiority. Transparent reporting should identify the nitrate and nitrite contributed by all sources, including natural ingredients, and should distinguish between their ingoing concentrations, residual concentrations and formation during processing.

In the United States, meat and poultry products are regulated principally by the US Department of Agriculture Food Safety and Inspection Service, with permitted curing agents and conditions of use specified in Title 9 of the Code of Federal Regulations and associated FSIS guidance [78]. Sodium or potassium nitrite may generally be used in cured meat products at levels that do not result in more than 200 ppm nitrite, calculated as sodium nitrite in the finished product, although product-specific requirements and restrictions apply. Sodium and potassium nitrate may be used as sources of nitrite in eligible cured meat products, including products requiring a longer maturation period. Ingredients employed as alternatives must also be safe and suitable for the intended purpose, and their use may be subject to FDA food-additive or generally recognised as safe provisions as well as FSIS requirements. These maximum concentrations are not directly equivalent to the revised EU limits because the two systems may differ in product classification, calculation basis and expression as sodium nitrite or nitrite ion [79,80]. A particularly important difference concerns US “uncured” labelling. Under 9 CFR §317.17 and §319.2, products conventionally associated with curing may be designated “uncured” when formulated without the nitrate or nitrite curing agents recognised under the applicable standard, provided that the required qualifying statements are displayed [78]. FSIS policy has historically applied this terminology to products manufactured using natural sources such as celery powder, even when those ingredients supply nitrate or nitrite and produce nitric oxide-mediated cured colour. Such products may therefore bear statements such as “uncured” or “no nitrate or nitrite added except for those naturally occurring in…” despite undergoing chemistry that is mechanistically similar to conventional curing. Conversely, a genuinely nitrate- and nitrite-free fermented product may be exempt from the accompanying “Not Preserved-Keep Refrigerated” statement where fermentation reduces the pH to 4.6 or below or drying reduces water activity to 0.92 or below. These thresholds constitute specific labelling provisions rather than universal demonstrations of microbial safety, and producers remain responsible for validating the complete manufacturing process. Thus, “uncured” should not be interpreted scientifically as synonymous with the absence of nitrate, nitrite, nitric oxide or nitrosylated pigments [78].

These frameworks have different implications for the six reformulation strategies considered above. Direct reduction remains governed by the permitted use level for conventional curing agents. Nitrate-rich vegetable ingredients require consideration of both their regulatory status and their contribution to total nitrate and nitrite exposure. Plant extracts intended to compensate for antioxidant or antimicrobial functions must be authorised or otherwise acceptable for the proposed ingredient, concentration and technological use. ZnPP-forming and bacterial nitric oxide synthase-positive cultures require strain-level safety assessment, technological validation and appropriate regulatory acceptance; in the case of bNOS, the absence of added nitrate or nitrite does not mean that nitric oxide-mediated curing chemistry is absent. Deliberately uncured products must meet general hygiene, food-safety and labelling obligations and cannot rely on the removal of curing salts as evidence of either safety or health benefit. Regulatory compliance must therefore be assessed for the complete ingredient–organism–process combination rather than inferred from the natural origin or historical food use of an individual component.

#### 2.1.7. Safety vs. Functionality

Reduction or elimination of nitrate and nitrite presents a multidimensional optimisation problem rather than a simple ingredient-substitution exercise. Lower use may reduce residual curing agents and potentially restrict conditions conducive to the formation of some *N*-nitroso compounds but may simultaneously weaken control of pathogenic and spoilage microorganisms, oxidative rancidity, colour deterioration and flavour instability. Conversely, retaining sufficient nitrite to provide technological protection does not eliminate the need to minimise exposure and control nitrosamine formation through raw-material quality, formulation, processing, storage and the use of appropriate inhibitors. The relevant objective is therefore not the lowest technically attainable nitrite concentration in isolation but the lowest concentration or alternative preservation system that maintains validated safety and acceptable product quality throughout manufacture and shelf life. No single alternative presently reproduces every function of nitrate and nitrite across all dry-fermented meat systems. Plant-derived curing changes the source of curing compounds without necessarily changing their chemistry; antioxidant and antimicrobial extracts provide selective functional compensation; ZnPP and bacterial nitric oxide synthase principally address pigment formation; and deliberately uncured reformulation redistributes preservation across fermentation, drying, cultures, formulation, packaging and storage. Each strategy consequently introduces its own trade-offs, including sensory changes, compositional variability, incomplete pathogen protection, altered oxidation, longer processing, new regulatory questions or increased dependence on cold-chain and packaging controls. Evidence of success for one endpoint such as increased redness, reduced TBARS or acceptable finished-product microbial counts must not be extrapolated to complete curing equivalence.

Future reformulation should therefore be guided by function-based validation. Studies should quantify nitrate and nitrite from all sources; distinguish ingoing, generated and residual concentrations; identify the pigment responsible for cured-like colour; assess lipid and protein oxidation; evaluate relevant pathogens and toxin formation under foreseeable processing and storage deviations; and include sensory acceptance and realistic shelf-life testing. Challenge organisms and performance criteria should be selected according to the intended product, process, packaging and storage conditions, with particular attention to *C. botulinum* where nitrite removal eliminates an established protective hurdle. Regulatory terminology should similarly reflect chemical and technological reality rather than imply that plant-derived or “uncured” products are necessarily nitrate-free, nitrite-free or intrinsically safer. Ultimately, the most credible route towards healthier and more sustainable fermented meats is unlikely to involve a universal replacement for nitrate and nitrite. It will instead require product-specific combinations of reduced curing-agent concentrations, selected microbial cultures, validated antimicrobial and antioxidant interventions, controlled acidification and drying, hygienic manufacture, appropriate packaging and evidence-based storage conditions. Innovation should be judged not by the complete disappearance of a familiar ingredient from the label but by whether the reformulated product achieves a demonstrably favourable balance among chemical exposure, microbiological safety, technological function, sensory acceptance and commercial feasibility.

### 2.2. Salt

Salt performs several interrelated technological functions in fermented meat manufacture, contributing to microbial stability, protein functionality, texture development and sensory quality. Dissolved NaCl increases the ionic strength of the meat matrix and promotes the swelling, solubilisation and extraction of salt-soluble myofibrillar proteins, particularly myosin. The extracted proteins support water and fat binding, particle adhesion and the development of a cohesive product structure; consequently, reducing NaCl can adversely affect water-holding capacity, binding and texture [81]. Salt also lowers water activity by increasing the concentration of dissolved solutes, thereby restricting the growth of many pathogenic and spoilage microorganisms and contributing to the selective conditions under which salt-tolerant fermentative and curing-associated microorganisms develop [82]. During fermentation and maturation, this effect operates alongside acidification by lactic acid bacteria and the progressive removal of moisture during drying. The resulting microbiological stability and product characteristics therefore reflect the combined influence of salt concentration, pH, water activity, starter-culture activity and processing conditions rather than the action of NaCl in isolation [83,84]. Salt further contributes directly to the characteristic taste of fermented meats and can influence fermentation behaviour, proteolysis, lipolysis, oxidative reactions and the formation or perception of flavour-active compounds [85,86,87]. Accordingly, reducing its concentration may have consequences extending beyond sodium content, and successful reformulation requires the preservation of microbial safety, physicochemical stability, structural integrity and sensory acceptability.

Despite its technological importance, reducing NaCl in fermented meat products remains a public health priority because habitual sodium intake above physiological requirements contributes to elevated blood pressure, a major modifiable risk factor for cardiovascular disease and stroke. The relationship between sodium intake and blood pressure is dose-responsive, and reductions in dietary sodium can lower blood pressure in both normotensive and hypertensive individuals, although the magnitude of the response varies among population groups [88,89,90]. This concern is particularly relevant to processed foods because much of the sodium consumed at the population level is incorporated during manufacture rather than added by consumers at the point of consumption [91]. Fermented meat products can contribute appreciably to this exposure because salt is conventionally used at concentrations required to support microbial stability, fermentation, drying, texture and flavour [92]. Accordingly, sodium reduction in this product category represents an important component of wider reformulation strategies. However, the nutritional benefit must be achieved without compromising product safety, technological performance or consumer acceptability, making fermented meats a particularly challenging target for salt reduction.

At the international level, the World Health Organization (WHO) recommends that adults consume less than 2 g sodium per day, equivalent to less than 5 g salt, and has called for a 30% relative reduction in mean population sodium intake by 2030 [89,93]. Within Europe, the European Commission established the EU Framework for National Salt Initiatives in 2008 to coordinate voluntary action by Member States, using a benchmark of at least a 16% reduction in salt levels over four years relative to national baseline values [94]. Ireland adopted an early national approach through the Food Safety Authority of Ireland (FSAI) Salt Reduction Programme, initiated in 2003, which combined engagement with the food industry, compositional monitoring and public health guidance [95]. The FSAI subsequently identified a mean population intake of 6 g salt per day as a pragmatic and achievable target for Irish adults while emphasising that this value should not be interpreted as an optimal level of consumption [96,97]. More recently, the Roadmap for Food Product Reformulation in Ireland established a target to reduce the salt content of priority food categories by 10% between 2015 and 2025, with implementation and monitoring undertaken by the Food Reformulation Task Force established in 2021 [98,99]. These initiatives place responsibility not only on consumers but also on food manufacturers to progressively reduce the sodium content of commonly consumed processed foods. Fermented and cured meat products are therefore important reformulation targets, although reductions must remain compatible with their microbiological safety, processing performance and characteristic sensory properties.

Long-term monitoring by the Food Safety Authority of Ireland indicates that voluntary reformulation has achieved measurable reductions in the sodium content of several processed-meat categories. Between 2004 and 2019, mean sodium concentrations decreased significantly by 12% in sausages, 24% in rashers and 17% in cooked ham, although no significant reduction was observed in traditional puddings [96]. These findings demonstrate that gradual sodium reduction is achievable within established commercial meat products, but the uneven progress among categories also highlights the product-specific constraints governing reformulation. Furthermore, sodium intake in Ireland remains above both the FSAI population target and the WHO recommendation despite an overall downward trend, indicating that continued reformulation and surveillance remain necessary [99]. Processed and cured meats retain relevance because of their contribution to dietary sodium exposure and the comparatively high concentrations of salt required in some products [96]. Further reductions cannot therefore be approached as simple compositional changes: the extent to which NaCl can be removed depends on its contribution to microbial control, protein functionality, fermentation, moisture loss, oxidative stability and sensory quality. Understanding the technological consequences of NaCl reduction is consequently essential to identifying reformulation strategies that deliver meaningful nutritional improvements without compromising product safety or acceptability.

Reducing NaCl can influence several interdependent processes during fermented meat manufacture because its effects extend across microbial ecology, protein functionality, moisture relations and biochemical reactions. A lower salt concentration may reduce the inhibition of salt-sensitive microorganisms and alter the competitive balance among lactic acid bacteria, coagulase-negative staphylococci, spoilage organisms and potential pathogens. Depending on the starter culture, formulation and processing conditions, this may modify the rate of acidification and the development of the characteristic fermentative microbiota [83,84,92]. Reducing NaCl may also increase water activity at an equivalent moisture content and weaken one component of the product’s combined preservation system. Its microbiological consequences must therefore be evaluated in relation to final pH, water activity, drying conditions, temperature, starter-culture performance and the presence of other antimicrobial hurdles rather than from salt concentration alone [84,100].

At the structural level, decreasing ionic strength can limit the extraction of salt-soluble myofibrillar proteins, potentially reducing water and fat binding, particle adhesion and the development of a cohesive texture [101]. Changes in salt concentration can also affect proteolysis, lipolysis, lipid and protein oxidation and the generation or perception of volatile compounds during fermentation and maturation [85,86,87]. These biochemical and structural effects may be expressed as changes in moisture loss, hardness, colour, aroma, taste and overall acceptability. Direct NaCl reduction can diminish perceived saltiness and modify characteristic aroma formation, while the use of alternative chloride salts may introduce bitterness, metallic notes or other sensory defects [83,85,102]. The magnitude and direction of these responses are product-specific and depend on the extent of NaCl reduction, the substitute employed, the starter culture and the wider formulation and processing system [84,103]. Consequently, a universally applicable minimum NaCl concentration or replacement strategy cannot be inferred across all fermented meats, with reformulation requiring specific validation of its microbiological safety, physicochemical stability and sensory acceptability.

Strategies for lowering sodium in fermented meats can be broadly divided into direct NaCl reduction and partial replacement with alternative salts. Direct reduction decreases the total chloride-salt concentration without introducing a substitute and therefore offers the simplest compositional approach. However, as the extent of reduction increases, insufficient ionic strength, diminished saltiness and weakened microbial inhibition may adversely affect protein extraction, water activity, fermentation, texture and flavour development [85,104,105]. Partial substitution seeks to retain more of the technological functionality of NaCl by replacing a proportion of it with other chloride salts. These approaches should be distinguished because reducing the total salt concentration and maintaining the chloride-salt concentration through ionic substitution can produce different physicochemical, microbiological and sensory outcomes.

Potassium chloride (KCl) is the most extensively investigated substitute because it can provide chloride ions and reproduce several of the ionic strength, water activity and protein functional effects of NaCl while lowering the sodium content of the formulation [102,103,106,107]. Its usefulness is nevertheless limited by sensory effects: increasing levels of replacement may reduce perceived saltiness or introduce bitter, metallic and otherwise atypical flavour notes. Consequently, partial rather than complete substitution is generally more feasible, although the acceptable replacement level varies according to product composition, processing conditions and the sensory characteristics of the fermented meat [84,102,106]. Calcium chloride (CaCl_2_) and magnesium chloride (MgCl_2_) have also been incorporated into mixed-salt systems, either alongside KCl or as additional NaCl substitutes. These divalent salts can contribute to sodium reduction but may affect protein functionality, texture, oxidation and flavour differently from both NaCl and KCl; excessive incorporation can likewise intensify bitterness or other undesirable sensory characteristics [86,87,105,108,109]. Their effectiveness therefore depends on the proportions used and the behaviour of the complete salt mixture rather than on sodium replacement alone.

Sensory-compensation strategies have been investigated to extend the degree of sodium reduction achievable without substantially impairing product acceptance. Yeast extract and combinations of lysine, taurine, disodium inosinate and disodium guanylate have been used to enhance savoury taste or mask defects associated with reduced NaCl and increased KCl [104,110]. Spices, aromatic plant extracts and other flavouring systems may serve a similar compensatory function, although they do not independently reproduce all of the antimicrobial and structural functions of NaCl [107]. Reformulation may therefore require a combined strategy in which partial NaCl replacement is integrated with flavour compensation, suitable starter cultures and appropriately controlled fermentation and drying. The outcomes reported for these approaches are summarised in Table 2, with emphasis on their technological, microbiological and sensory effects in the specific products investigated.

Collectively, the studies summarised in Table 2 demonstrate that meaningful sodium reduction can be achieved in fermented meat products, but no single strategy consistently preserves all the functions provided by NaCl. Moderate direct reduction appears feasible in some formulations, whereas more extensive reduction can alter water activity, acidification, microbial ecology, texture and flavour development. Partial replacement with KCl generally retains technological functionality more effectively than direct NaCl removal, although increasing substitution can introduce bitterness, metallic notes and changes in characteristic aroma. Mixed chloride-salt systems and flavour-compensation ingredients may extend the achievable degree of replacement, but their effects depend strongly on the composition of the substitute mixture and the product matrix. The available evidence therefore supports carefully optimised partial substitution or combined reformulation strategies rather than the complete removal or universal replacement of NaCl.

The increased use of potassium-based salt substitutes also requires appropriate nutritional and risk-management consideration. For most healthy individuals, dietary potassium is physiologically regulated, and replacing part of the sodium chloride with KCl may improve the sodium-to-potassium balance of the product [112,113]. However, individuals with impaired potassium excretion or regulation, including some people with renal or cardiovascular conditions and those taking medications that increase serum potassium, may be susceptible to hyperkalaemia and may have been advised to restrict potassium intake [114,115]. The use of KCl should therefore be supported by a product-specific risk assessment, consideration of the quantity and frequency with which the food is consumed, compliance with applicable compositional and labelling requirements and clear communication of potassium content where necessary [116,117]. These considerations do not preclude the use of KCl but emphasise that its suitability must be assessed from both technological and nutritional perspectives.

Ultimately, successful sodium reduction in fermented meats requires reformulation at the level of the complete preservation system rather than the simple removal of a single ingredient. Candidate formulations should be validated under representative manufacturing and storage conditions to determine their effects on fermentation kinetics, starter-culture performance, pathogen and spoilage control, pH, water activity, drying behaviour, protein functionality, oxidative stability and sensory acceptability. Attention should be given to process variability and to conditions representing the least favourable combination of preservation hurdles. Future research should also report both the degree of NaCl replacement and the resulting sodium and potassium contents, as percentage replacement alone does not establish the nutritional effect of reformulation. Integrating partial salt substitution with suitable starter cultures, flavour-compensation approaches and controlled processing may therefore provide the most effective route towards fermented meat products that contain less sodium while retaining their safety, quality and characteristic sensory properties.

## 3. Smoking

Smoking is a long-established processing operation in the manufacture of many dry- and semi-dry-fermented meat products, where it contributes to characteristic flavour, aroma, colour and product identity while complementing the preservative effects of fermentation, curing and drying [118,119,120,121]. Wood smoke contains a complex mixture of volatile compounds, among which phenolic compounds, carbonyls and organic acids are particularly important to product quality. Phenolic compounds contribute characteristic smoky aromas and may exert antioxidant and antimicrobial effects, while carbonyl compounds participate in surface colour development and provide additional aroma notes [120,122]. The composition and deposition of these compounds depend on factors including the wood species, smoke-generation conditions, smoking duration, airflow and product surface characteristics [123,124]. Smoking may also promote limited surface dehydration; however, the final water activity and microbiological stability of fermented meat products result from the combined effects of formulation, acidification, curing and progressive drying rather than smoking alone [121]. Accordingly, smoking should be regarded as one component of the wider hurdle system used in fermented meat manufacture, rather than as an independent preservation treatment.

The sensory character imparted by smoking reflects both the deposition of smoke constituents onto the product surface and their subsequent interaction with the meat matrix. Phenolic compounds provide many of the characteristic smoky, woody and spicy aroma notes, while carbonyl compounds contribute to aroma and participate in reactions with amino groups at the product surface that support the development of the characteristic smoked colour. Organic acids and other low-molecular-weight constituents may provide additional flavour notes and contribute to the antimicrobial activity of smoke, although their effects depend on their concentration and the conditions under which smoking is performed [120]. Smoke-derived compounds may also interact with volatile substances generated through fermentation, lipid oxidation, proteolysis and seasoning, producing a product-specific aroma profile rather than a flavour attributable to smoke alone [118,119,125]. Their deposition and penetration are not uniform, with concentrations generally greatest at the product surface, and are influenced by smoking duration, casing properties and the physicochemical characteristics of individual compounds [126,127]. Consequently, smoking intensity must be controlled to achieve the desired sensory character without producing excessive surface deposition or undesirable bitter, acrid or ashy notes. This control is also important because the thermal decomposition of wood can generate undesirable compounds alongside the constituents responsible for flavour, colour and preservation, most notably polycyclic aromatic hydrocarbons (PAHs) [128,129].

Polycyclic aromatic hydrocarbons (PAHs) are generated through the incomplete combustion and pyrolysis of organic material during smoke production and may subsequently be transported in the particulate and vapour phases of smoke before being deposited onto the product surface [128,129,130]. Their formation and accumulation in smoked meats are highly process-dependent and are influenced by the type and condition of the fuel, smoke-generation temperature, oxygen availability, combustion efficiency, smoke density, airflow, smoking duration and the distance between the smoke source and the product [123,131]. Direct exposure to combustion gases, contact with flames, and the deposition of soot or tar particles can further increase contamination, whereas indirect smoke generation and controlled delivery provide greater regulation of smoke composition and product exposure [132]. Importantly, the temperature at which wood undergoes pyrolysis or combustion should be distinguished from that maintained within the smoking chamber. Smoke is commonly generated through fuel pyrolysis at approximately 300–450 °C in the glow zone, whereas the chamber temperature employed for cold smoking is substantially lower [130]. Although chamber conditions can affect smoke deposition and exposure duration, PAH generation is principally governed by conditions at the smoke source and by the subsequent transfer of contaminants to the product [131]. Product characteristics, including fat content, casing permeability, surface moisture and surface area, may additionally affect PAH retention and penetration [126,128]. Consequently, PAH concentrations can vary substantially among smoked fermented meats according to the equipment and process employed, and smoking should not be treated as a uniform source of contamination [132,133,134,135].

The process-dependent nature of PAH formation is particularly important when interpreting their occurrence in smoked meat products. Modern smoking systems employing controlled smoke generation, regulated airflow and temperature, indirect smoke delivery, appropriate fuel materials and measures to limit the transfer of soot and tar can maintain PAH concentrations well below the applicable maximum levels when correctly operated and validated [136,137]. Smoked meats should therefore not be characterised generally as foods containing particularly high PAH concentrations. Elevated concentrations are more commonly associated with poorly controlled smoking conditions, including direct exposure to combustion gases or flames, use of unsuitable or excessively moist fuel, inefficient combustion, inadequate separation between the smoke source and product and prolonged or repeated smoking without effective process regulation [123,130,133,134]. Such conditions may occur in domestic or small-scale traditional production where smoking parameters and equipment are not standardised; however, traditional manufacture should not itself be regarded as inherently unsafe, as PAH concentrations can be substantially reduced through optimisation of the smoking procedure while preserving the characteristic sensory identity of the product [136]. Conversely, the use of modern equipment does not remove the need for process validation, routine maintenance and analytical verification. PAH risk should therefore be evaluated according to the specific fuel, smoke-generation system, equipment and operating conditions employed, rather than attributed indiscriminately to smoked meat products as a category [126,138].

Cold smoking is the principal smoking approach used in the manufacture of dry-fermented meat products because it allows smoke-derived flavour, aroma and colour to develop without cooking the product or disrupting its raw-ripened characteristics [121,137]. Codex classifies cold smoking as occurring at chamber temperatures of approximately 18–25 °C and specifically identifies salami-type sausages among the products manufactured using this process [130]. This range provides a useful technological reference point rather than a universally fixed definition, since the precise temperature, duration and frequency of smoke exposure vary according to product type, regional manufacturing practice and the stage of fermentation or drying at which smoking occurs [139]. Repeated or intermittent smoking may contribute to surface drying and influence the development of the product volatilome and microbial ecology; however, these effects depend on the intensity and conditions of the process and should not be interpreted as providing an independent preservation or pathogen-inactivation treatment [140]. Cold smoking does not deliver the thermal lethality associated with cooking and cannot compensate for inadequate control of raw-material hygiene, fermentation, acidification, curing, water activity or drying [141]. Its safe application therefore depends on integration within a validated hurdle system in which starter-culture activity, salt and curing ingredients, pH reduction, moisture removal and hygienic process control collectively restrict the survival or growth of pathogenic and spoilage microorganisms [8]. Cold-smoking conditions must consequently be optimised not only to achieve the required sensory profile but also to avoid disrupting fermentation and drying or introducing excessive PAH contamination [136,137,139].

Effective control of PAHs requires optimisation of the complete smoking process rather than reliance on any single corrective measure. The principal preventive strategies include the use of clean, untreated and appropriately seasoned wood; separation of smoke generation from the smoking chamber; avoidance of direct flame contact; control of pyrolysis conditions, oxygen supply and airflow; and limitation of soot and tar transfer through suitable smoke-cleaning, filtration or deposition systems [138,142]. Smoking duration, frequency and smoke density should also be adjusted to the minimum levels required to achieve the desired sensory characteristics, as excessive exposure can increase surface accumulation of PAHs without providing a corresponding technological benefit [131]. Fraqueza et al. demonstrated that this principle was effective in traditional dry-cured sausages for which a cold-smoking period of 20 h, distributed across three days, maintained the required sensory characteristics while concentrations of benzo[a]pyrene and PAH4 remained below their respective maximum levels [136]. Although total PAH concentrations did not differ significantly among the smoking regimes investigated, which ranged from no smoking to 60 h of effective exposure or smoking until 38–40% weight loss, the authors recommended limiting cold smoking to a maximum of 20 h as a product-specific optimisation measure. Adequate distance between the smoke source and product, regular removal of combustion residues and appropriate cleaning and maintenance of smoke generators, ducts and chambers further reduce the potential for contamination [134,140]. Because PAHs are deposited predominantly at the product surface, casing selection and removal may influence consumer exposure in products for which the casing is not intended to be eaten; however, casing-based mitigation cannot replace control of PAH formation at its source [126,127]. Process modifications must additionally be evaluated against their effects on flavour, colour, drying behaviour and microbiological stability, since an intervention that reduces smoke exposure may also alter product identity or weaken a contributory preservation hurdle [138]. Compliance should therefore be supported by product-specific process controls and routine monitoring of BaP and PAH4 in accordance with the applicable regulatory requirements [143].

Benzo[a]pyrene (BaP) is a genotoxic and carcinogenic PAH that has historically been used as a marker of PAH contamination in food. However, its absence or occurrence at a low concentration does not necessarily indicate the corresponding absence of other toxicologically relevant PAHs [144]. The European Food Safety Authority therefore concluded that BaP alone is not a suitable indicator of PAH occurrence and toxicity and identified PAH4 as a more representative marker [145]. PAH4 comprises the sum of benzo[a]pyrene, benz[a]anthracene, benzo[b]fluoranthene and chrysene and is now assessed alongside BaP in European Union contaminant legislation. Under Commission Regulation (EU) 2023/915, smoked meat and smoked meat products are subject to maximum levels of 2.0 μg/kg for BaP and 12.0 μg/kg for PAH4 [143]. For PAH4, the regulatory value is expressed as a lower-bound concentration, whereby the concentrations of any of the four compounds falling below the analytical limit of quantification are treated as zero [143]. The Regulation permits specified Member States to authorise certain traditionally smoked meat products manufactured and marketed within their own territories at higher maximum levels of 5.0 μg/kg for BaP and 30.0 μg/kg for PAH4, provided monitoring continues and good smoking practices are implemented where possible without loss of the products’ characteristic organoleptic properties [143]. These derogations, which include traditionally smoked meat and meat products produced in Ireland, are restricted exceptions and should not be interpreted as alternative general limits for smoked fermented meats.

Smoke flavourings were developed as alternatives or complements to direct smoking and were intended to impart smoke-derived sensory or technological properties without direct exposure of the food to conventionally generated smoke. Their manufacture begins with the controlled generation and condensation of wood smoke, followed by physical separation of the condensate into a water-based primary smoke condensate, a water-insoluble high-density tar phase and an unusable oily phase. The primary smoke condensate and appropriately processed fractions of the high-density tar phase, termed primary tar fractions, are subsequently purified to remove undesirable constituents and may be used directly or further processed to produce derived smoke flavourings [146]. In meat manufacture, smoke flavourings may be incorporated into the formulation or applied to the product surface, depending on the product and process. Their use avoids some of the variability associated with direct smoke generation, in which wood type, combustion conditions, airflow, smoking time and smokehouse operation influence product characteristics and PAH formation [128]. Smoke flavourings may additionally provide antioxidant and antimicrobial effects, although the magnitude of these effects depends on the composition, concentration and application of the individual preparation and cannot be assumed to reproduce the complete preservative contribution of conventional smoking [128,147].

The regulatory position of smoke flavourings in the European Union changed following EFSA’s 2023 reassessment of the eight smoke flavouring primary products for which renewal applications had been submitted. Applying its updated approach for evaluating complex mixtures, EFSA concluded that genotoxicity concerns could not be excluded for any of the eight products: six contained substances for which genotoxicity concerns were identified, while the available evidence was insufficient to exclude such concerns for the remaining two [148,149,150,151,152,153,154,155]. This conclusion represents the identification of a potential hazard and should not be interpreted as demonstrating that consumption of every food containing these preparations necessarily causes adverse health effects, since EFSA did not assess the likelihood of harm arising from individual dietary exposure [156]. The authorisations of the eight reassessed products (SF-001 to SF-006, SF-008 and SF-009) were subsequently not renewed, while no renewal applications had been submitted for SF-007 and SF-010. Commission Implementing Regulation (EU) 2024/2067 accordingly deleted entries SF-001 to SF-010 from the Union list of authorised smoke flavouring primary products and established transitional measures intended to allow manufacturers time to reformulate affected foods [157]. For the eight reassessed products, foods in category 8, encompassing meat and its corresponding subcategories, that complied with the previously applicable conditions may continue to be placed on the market until 1 July 2029 and may remain available thereafter until their date of minimum durability or “use by” date. The corresponding deadline for placing foods in most other categories on the market was 1 July 2026; products lawfully placed on the market by that date may remain available until their date of minimum durability or “use by” date [157]. The regulatory change should therefore be described as the non-renewal of the relevant authorisations followed by category-specific transitional phase-out periods, rather than as an immediate or universal prohibition of smoke-flavoured foods.

The non-renewal of smoke flavouring authorisations creates a multifaceted reformulation challenge for fermented meat manufacturers because these preparations contributed not only smoky flavour and aroma but also colour development, oxidative stability, microbial inhibition and process standardisation. No single replacement technology can be assumed to reproduce all functions, and reformulation should therefore begin by identifying the specific sensory and technological roles previously provided by the smoke flavouring in each product [128,142,147]. Where retention of an authentic smoked character is essential, carefully controlled conventional cold smoking represents the most direct established option, if smoke generation, fuel selection, airflow, exposure time and PAH deposition are validated in accordance with good smoking practices [130,136]. Indirect smoke generation, effective separation of the product from the combustion source, smoke cleaning or filtration and precise control of smoke density may allow manufacturers to achieve the required sensory profile while limiting soot, tar and PAH transfer [132]. Product-specific optimisation will nevertheless be necessary because reduced smoke exposure or altered smoke composition may affect colour, aroma, lipid stability, surface drying and microbial ecology [137,138]. Where direct smoking is unsuitable or undesirable, its former functions may instead need to be reconstructed through a combination of permitted flavouring ingredients, antioxidants, antimicrobial cultures or compounds and validated processing and packaging hurdles; however, such measures should be described as complementary reformulation tools rather than as direct replacements for smoking unless their sensory and technological equivalence has been demonstrated in the target fermented meat product [121]. The regulatory transition therefore reinforces the need for integrated, product-specific reformulation supported by sensory assessment, microbiological validation, oxidative-stability testing and analytical verification of PAH compliance.

## 4. Fat Replacement in Fermented Meat Products

Traditional fermented sausages commonly contain substantial quantities of animal fat, principally pork backfat, which contributes to their energy density and saturated fatty acid (SFA) content [158,159]. Consequently, increasing consumer interest in products with more favourable nutritional profiles has encouraged the development of reformulation strategies aimed at reducing total fat, decreasing SFA content and increasing the relative proportions of monounsaturated and polyunsaturated fatty acids (MUFAs and PUFAs, respectively) [158,160] This objective is consistent with current World Health Organization guidance that dietary fat should be derived primarily from unsaturated fatty acids and that SFAs should provide no more than 10% of total energy intake [161]. Fat reformulation should nevertheless be understood as an improvement in product composition rather than evidence that the resulting fermented meat product is intrinsically “healthy” or confers a direct health benefit. Moreover, because animal fat performs essential structural, technological and sensory functions during fermented sausage manufacture, its reduction or replacement must be carefully balanced against the requirements for successful processing and acceptable finished-product quality.

Pork backfat is not merely a source of lipid but a technologically functional component of dry-fermented sausages. When sufficiently firm and maintained at an appropriate processing temperature, backfat can be comminuted into discrete particles that contribute to the characteristic appearance, texture, flavour release and mouthfeel of the finished product [158,160]. During mixing, salt-solubilised myofibrillar proteins contribute to the formation of a cohesive meat matrix, which consolidates as fermentation, acidification and drying progress [158,162]. Excessively soft or oily fat, which may result from a high degree of fatty acid unsaturation or inadequate temperature control, is more susceptible to deformation and smearing during comminution [160,163]. Released or insufficiently immobilised lipid may disrupt matrix binding and interfere with moisture removal, thereby compromising product structure and firmness and, consequently, sliceability [162,164]. Consequently, successful fat reformulation depends not only on the quantity and fatty acid composition of the replacement lipid but also on its physical state, particle stability and behaviour throughout grinding, fermentation and ripening [160,162,164,165].

Fat reformulation strategies in fermented sausages can be broadly differentiated according to whether they reduce the quantity of fat, replace animal fat during manufacture or modify the composition of the animal fat before processing. The simplest approach involves reducing pork backfat without direct substitution, although substantial reductions may adversely affect processing yield, texture, mouthfeel and sensory acceptance. Alternatively, part of the backfat may be replaced with non-lipid ingredients, such as dietary fibres or protein- and carbohydrate-based matrices, which can provide bulk, bind water and partially reproduce some of the structural functions of fat [158]. A further strategy involves replacing backfat with vegetable- or marine-derived oils to decrease SFA content and increase MUFA and/or PUFA proportions. As liquid oils do not possess the physical properties required of solid backfat, they are commonly incorporated within structured delivery systems, including pre-emulsions, gelled emulsions, oleogels and polysaccharide-based matrices [164,165,166,167,168,169]. These systems are intended to immobilise the oil, improve its distribution and facilitate its incorporation into the developing sausage matrix. However, their effectiveness depends on the structuring material, lipid composition, replacement level and processing conditions. Distinct from these ingredient-level interventions, pre-harvest strategies can alter the fatty acid composition of porcine adipose tissue through dietary modification, thereby retaining animal fat as the structural lipid component while modifying its nutritional profile [170,171]. Each approach therefore presents a different balance among fat reduction, fatty acid modification, processing functionality, oxidative stability and sensory quality.

Among these approaches, structured oil systems have received particular attention because they enable unsaturated oils to be incorporated in a semi-solid form that more closely approximates the processing behaviour of animal fat. Emulsion gels immobilise oil droplets within protein- or polysaccharide-based networks, whereas oleogels convert liquid oils into self-supporting structures through the formation of a three-dimensional gel network. Their effectiveness nevertheless depends on both the oil composition and the method used to structure it. In heat-treated fermented beef sausages, Öztürk-Kerimoğlu et al. [165] used cold- and hot-set emulsion gels containing a blend of predominantly MUFA-rich peanut oil and PUFA-rich linseed oil, the latter providing α-linolenic acid. Reformulation decreased the SFA proportion from 46.6% to 23.5% and increased MUFAs and PUFAs from 47.3% to 51.0% and from 4.7% to 25.4%, respectively. However, gelation method influenced product performance: cold-set gels provided greater PUFA retention and produced fewer colour changes, whereas hot-set gels yielded a more favourable microstructure, lower purge loss and improved sensory scores [165]. In dry-fermented sausages, linseed oil has similarly been incorporated within carrageenan-based gelled emulsions [166], while inulin-based emulsion-filled gels have simultaneously facilitated partial backfat replacement, PUFA enrichment and the addition of prebiotic fibre [168]. More complex systems containing olive, linseed and fish oils stabilised in a konjac matrix achieved substantial reductions in total fat and SFA content, although the accompanying decreases in sensory scores demonstrating that compositional improvement did not necessarily preserve product acceptance [167]. Oleogelation offers an alternative means of structuring unsaturated oils: replacement of 50% of pork backfat with an olive oil oleogel produced fermented sausages with lower SFA and cholesterol contents, while the oleogel-only formulation retained sensory characteristics most comparable to those of the control [169]. Collectively, these studies demonstrate that structured oil systems can improve the lipid composition of fermented sausages, but their technological and sensory success remains dependent on the lipid source, structuring matrix, gelation method and degree of backfat replacement.

Increasing the level of fat substitution can introduce substantial technological and sensory limitations even where the intended reduction in total fat or SFA content is achieved. Successful reformulation requires the replacement phase to remain sufficiently immobilised and compatible with the meat matrix throughout comminution, fermentation and drying. Where this is not achieved, oil agglomeration or release may weaken matrix cohesion, alter moisture migration and drying behaviour and produce softer products with poorer structural integrity. These effects are strongly dependent on the replacement material and its concentration. For example, pre-emulsified olive oil retained acceptable sensory characteristics when replacing up to 25% of pork backfat in Chorizo de Pamplona, whereas higher substitution levels resulted in unacceptable oil release during ripening [164]. Similarly, the direct incorporation of olive oil into dry-cured fermented sausages produced inadequate oil retention, impaired matrix binding, reduced drying losses and a softer, technologically unacceptable structure [162]. Structured systems can alleviate some of these difficulties but do not eliminate them: carrageenan-based linseed oil emulsions produced formulation-dependent changes in texture and sensory properties [166], while the replacement of backfat with an olive–linseed–fish oil mixture stabilised in a konjac matrix substantially reduced total fat but decreased the scores of all sensory attributes assessed [167]. Conversely, as summarised in Table 3, other systems maintained comparatively acceptable sensory quality at the replacement levels investigated [165,168,169], demonstrating that adverse effects are not an inevitable consequence of reformulation. Rather, technological feasibility and consumer acceptance depend on the interaction among lipid composition, physical structure, substitution level and product-specific processing conditions. Replacement levels should therefore be optimised experimentally rather than interpreted as universally applicable thresholds.

**Table 3:** Selected fat reformulation strategies in fermented meat products and their compositional, technological and sensory outcomes.

Reformulation Strategy	Replacement Material/System	Product and Substitution Level	Principal Compositional Effects	Technological, Oxidative and Sensory Outcomes	Ref.
Pre-emulsified oil	Pre-emulsified olive oil	Chorizo de Pamplona; graded replacement of pork backfat	Improved fatty acid profile through partial replacement of animal fat with olive oil.	Sensory acceptability was retained at replacement levels up to 25%; higher levels caused unacceptable oil release during ripening, demonstrating a product-specific technological limit.	[164]
Inulin-based gelled suspension or emulsion	Inulin gelled suspension or inulin–linseed-oil emulsion-filled gel	Dry-fermented sausage; 16% replacement of pork backfat	Fat content decreased from 44.37% in the control to 31.38% and 35.36% in sausages containing the inulin suspension and emulsion-filled gel, respectively. The linseed oil system increased PUFAs and improved the *n*-6/*n*-3 ratio.	Modified products exhibited lower hardness, chewiness and springiness and greater adhesiveness but remained sensorially acceptable. The linseed oil formulation showed greater susceptibility to oxidation and lipolysis.	[168]
Peanut–linseed-oil emulsion gel	Cold- or hot-set gelled emulsions containing predominantly MUFA-rich peanut oil and PUFA-rich linseed oil	Heat-treated fermented beef sausage; partial replacement of beef fat	SFAs decreased from 46.6% to 23.5%, while MUFAs increased from 47.3% to 51.0% and PUFAs from 4.7% to 25.4%. Nutritional lipid indices were also improved.	Cold-set gels produced fewer colour changes and greater PUFA retention; hot-set gels provided a more favourable microstructure, lower purge loss and higher sensory scores.	[165]
Fig-seed-oil gelled emulsion	Fig-seed-oil-in-water gelled emulsion	Fermented beef sausage; 50% or 100% replacement of beef fat	Replacement altered the lipid source without increasing TBARS in the 100%-replacement treatment.	The 50%-replacement treatment exhibited the highest TBARS values, and oxidation increased during storage. Sensory attributes nevertheless remained within acceptable ranges.	[172]
Carrageenan-based gelled emulsion	High-*n*-3 linseed oil emulsion structured with carrageenan	Dry-fermented sausage; 26.3%, 32.8% or 39.5% pork backfat substitution	PUFA content increased to a maximum of approximately 10.3%.	Textural and sensory effects depended on substitution level. At 32.8% substitution, colour and sensory characteristics remained comparable to the control; only limited formation of lipid-oxidation-derived volatile aldehydes was observed.	[166]
Polysaccharide-stabilised oil matrix	Olive, linseed and fish oil mixture immobilised in a konjac glucomannan matrix	Dry-fermented sausage; replacement system producing low-fat formulations	Total fat decreased by 58–68%, from 316 g/kg in the control to 99–130 g/kg in reformulated products. SFAs decreased, PUFAs increased, and the *n*-6/*n*-3 ratio improved.	Fat reduction decreased hardness and increased cohesiveness, without significantly affecting springiness or chewiness. All evaluated sensory attributes received lower scores than the control.	[167]
Direct incorporation of liquid oil	Olive oil incorporated with or without high-pressure-processed meat as a fat replacer	Dry-cured fermented sausage	Reduced animal-fat content through incorporation of an unsaturated plant oil.	Inadequate oil immobilisation caused oil agglomeration and release, impaired matrix binding and water evaporation, reduced drying losses and produced a softer, technologically unacceptable structure.	[162]
Oleogel	Olive oil oleogel, evaluated independently and with partial NaCl replacement by KCl	Fermented sausage; 50% replacement of pork backfat, with or without simultaneous 50% NaCl replacement	Simultaneous fat and salt reformulation reduced sodium by 51.9%, cholesterol by 19.2% and SFAs by 17.8% relative to the control.	The formulation in which 50% of pork backfat was replaced by oleogel produced characteristics most comparable to those of the control; simultaneous salt and fat reformulation introduced additional product-quality effects.	[169]

Abbreviations: KCl, potassium chloride; MUFAs, monounsaturated fatty acids; NaCl, sodium chloride; PUFAs, polyunsaturated fatty acids; SFAs, saturated fatty acids; TBARS, 2-thiobarbituric acid-reactive substances.

Comparable technological constraints apply when the fatty acid composition of animal fat is modified before manufacture because increasing its degree of unsaturation can alter the physical and oxidative properties of the adipose tissue itself. Dietary modification of finishing pigs provides a pre-harvest alternative to replacing backfat during sausage manufacture, enabling the fatty acid profile of porcine adipose tissue to be altered while retaining animal fat as the structural lipid phase [171]. However, MUFA and PUFA enrichment should not be considered technologically equivalent. Increasing MUFAs, particularly oleic acid, may improve the unsaturated-to-saturated fatty acid ratio while preserving greater fat firmness than extensive PUFA enrichment. By contrast, increasing PUFA content generally alters fat melting characteristics and may produce softer adipose tissue that is more susceptible to deformation and smearing during comminution, with possible consequences for particle definition, matrix formation and product texture [160,171]. Rubio et al. demonstrated the feasibility of producing dry-fermented *Salchichón* from raw materials enriched separately in MUFAs or PUFAs [170]. However, the resulting products required evaluation throughout modified-atmosphere storage because the altered lipid composition also influenced their susceptibility to quality deterioration. Pre-harvest modification must therefore be considered in relation to the technological suitability of the resulting fat rather than solely the magnitude of fatty acid enrichment achieved. In particular, the degree and type of unsaturation should be optimised to preserve sufficient fat firmness for processing while achieving the intended nutritional modification.

Increased unsaturation also has important implications for oxidative stability, because PUFAs contain multiple double bonds that increase their susceptibility to lipid oxidation during processing and storage [159,160]. This vulnerability is particularly relevant in fermented sausages, where comminution increases lipid exposure to oxygen and the prolonged fermentation, drying and storage periods provide opportunities for oxidative reactions to develop. Oxidation may generate undesirable flavours and odours, impair colour and reduce the retention of nutritionally valuable unsaturated fatty acids. Consequently, lipid reformulation should be evaluated using measures of oxidative stability alongside fatty acid composition, technological performance and sensory quality [159,160]. The magnitude of this effect is nevertheless formulation-dependent and may be moderated through appropriate lipid structuring, antioxidant protection, processing control and oxygen-limiting packaging. For example, Rubio et al. evaluated MUFA- and PUFA-enriched *Salchichón* under modified-atmosphere storage, illustrating the importance of considering the stability of reformulated products throughout their intended shelf life rather than solely at the completion of manufacture [170]. Collectively, the available evidence demonstrates that reductions in total fat or SFA content and increases in MUFAs or PUFAs can improve selected compositional characteristics of fermented meat products, but these changes cannot alone establish an overall nutritional or technological advantage. Successful reformulation requires simultaneous optimisation of fatty acid composition, fat-phase structure, matrix compatibility, drying behaviour, oxidative stability and sensory acceptance. Accordingly, the most promising strategies are not necessarily those achieving the greatest degree of fat replacement or unsaturated-fatty-acid enrichment but those that deliver a meaningful compositional improvement while preserving product safety, stability, processing performance and consumer acceptability.

## 5. Microbiology of Fermentation and Microbial Ingredients

Microorganisms perform multiple and interdependent functions during fermented meat manufacture, influencing acidification, microbial stability, colour, flavour, texture and overall product consistency. Although these activities may arise from the indigenous microbiota during spontaneous fermentation, commercial manufacture commonly employs selected starter cultures to produce more predictable fermentation dynamics and finished-product characteristics. Lactic acid bacteria principally contribute through carbohydrate fermentation and organic acid production, thereby lowering the pH and restricting the growth of susceptible undesirable microorganisms, whereas coagulase-negative staphylococci and other technologically relevant microorganisms may support colour development, oxidative stability and the formation of characteristic flavour compounds through strain-dependent enzymatic activities [8,41]. Accordingly, starter-culture selection increasingly extends beyond reliable fermentation initiation towards the use of microorganisms possessing specific technological, protective or functional properties. This development has created opportunities to design multifunctional culture systems that may compensate for selected preservative reductions while maintaining product quality and microbial control.

Beyond their primary roles in fermentation, selected microorganisms may provide complementary functions that support colour development and microbial control in products manufactured with reduced concentrations of conventional curing agents. Coagulase-negative staphylococci have established curing-associated roles, while selected lactic acid bacteria have also demonstrated strain-dependent colour-forming and antimicrobial activities in experimental meat systems [41,59,173,174]. For example, selected *Lactiplantibacillus plantarum* isolates produced red myoglobin derivatives and exhibited antimicrobial activity, alongside enzymatic activities associated with biologically mediated colour formation [174]. Similarly, *Lactiplantibacillus pentosus* has been investigated in fermented sausage as a component of partial sodium nitrite-replacement strategies [175]. These findings identify microbial cultures as potential contributors to multifunctional curing systems; however, evidence obtained through strain screening or model meat systems does not independently demonstrate complete replacement of the technological and antimicrobial functions of nitrite. Such cultures should therefore be regarded as complementary components of validated hurdle systems rather than direct, universally applicable nitrite substitutes. The nitric oxide- and Zn-protoporphyrin IX-mediated pathways underlying alternative colour formation are discussed in detail in Section 2.1.

The expanding range of microbial applications in fermented meat manufacture nevertheless requires careful distinction between technological suitability and substantiated functional effects. Conventional starter cultures are selected principally to initiate and regulate fermentation and to promote predictable safety and quality characteristics, whereas other cultures may be investigated for additional protective or host-directed functions [4,41,176,177]. A single strain may possess several potentially useful traits; for example, it may tolerate sausage-processing conditions, contribute to acidification and inhibit selected undesirable microorganisms. However, demonstrating these technological properties does not independently establish that the strain is probiotic or that consumption of the resulting product will confer a health benefit. Probiotic designation requires precise strain characterisation, delivery of viable microorganisms in an adequate amount and evidence of a strain-specific benefit in the host [4,178]. Accordingly, microorganisms that perform successfully during fermented meat manufacture should be described as starter cultures, protective cultures or probiotic candidates according to their intended application and level of substantiation, rather than treating these classifications interchangeably.

### 5.1. Probiotics and Prebiotics

Fermented foods, probiotics and prebiotics represent related but conceptually distinct categories. Fermented foods are produced through desired microbial growth and enzymatic conversion of food components; however, fermentation alone does not establish probiotic functionality, and some fermented products may no longer contain viable microorganisms when consumed [4]. Probiotics are live microorganisms that confer a demonstrated health benefit on the host when administered in adequate amounts, meaning that the designation depends on strain identity, viable delivery and evidence of benefit rather than the presence of live cultures alone [178]. By contrast, a prebiotic is a substrate that is selectively utilised by host microorganisms and thereby confers a health benefit [179]. Consequently, a fermented meat product should only be described as probiotic when it delivers an adequately characterised microorganism for which an appropriate host benefit has been substantiated, while incorporation of a fermentable ingredient does not establish prebiotic status unless selective microbial utilisation and an associated health benefit have also been demonstrated [4,178,179].

Substantiation of probiotic functionality therefore requires several complementary forms of evidence. The microorganism must be identified at the strain level using appropriate phenotypic and molecular methods, since safety, technological performance and health effects cannot necessarily be extrapolated between strains of the same species [4,178]. Its safety must also be evaluated in relation to the intended population and application, including relevant virulence properties, acquired antimicrobial-resistance determinants and other strain-specific hazards. The organism must remain viable at a quantitatively adequate level throughout manufacture and storage and be delivered in the portion of food consumed, requiring enumeration within the finished product rather than the inoculum alone [180]. However, no single viable count constitutes a universal probiotic dose, as the effective amount depends on the strain, product, intended benefit and supporting efficacy studies [178,180]. Finally, survival under processing conditions, tolerance of simulated gastrointestinal environments or recovery following consumption may demonstrate technological suitability and delivery potential, but these findings do not independently establish a health benefit. Probiotic designation ultimately requires appropriately designed host studies linking consumption of the defined strain, at the delivered dose, to a substantiated beneficial outcome [4,178].

Fermented meat products have consequently been investigated as potential carrier matrices for appropriately characterised probiotic strains, particularly as non-dairy alternatives that may broaden the range of foods through which live microorganisms can be delivered [176,177]. Their suitability depends on compatibility between the selected strain, product formulation and manufacturing process. Candidate cultures must remain metabolically and technologically compatible with the indigenous microbiota or conventional starter system while tolerating salt, curing agents, acidification, drying, reduced water activity, refrigeration and prolonged storage without adversely affecting product safety or sensory quality [176,177,181,182,183]. The meat matrix may offer some protection during gastrointestinal transit, and products that receive no lethal treatment after fermentation may permit viable microorganisms to remain in the food until consumption. Nevertheless, these potential advantages cannot be assumed across fermented meat products because processing intensity, formulation, storage conditions and strain-specific robustness substantially influence survival. Technological screening should therefore evaluate growth and acidification kinetics, tolerance of relevant processing stresses, interactions with established starter cultures, antagonism towards undesirable microorganisms, production of biogenic amines or other unwanted metabolites and effects on colour, flavour and texture [176,177,181,182,183]. Viable counts should subsequently be monitored throughout manufacture and the declared shelf life at the level of the finished product to establish whether the intended dose is realistically delivered at consumption [180]. The nature and evidential strength of selected studies evaluating fermented sausages as carriers of candidate probiotic microorganisms are summarised in Table 4.

**Table 4:** Selected studies evaluating fermented sausages as carriers of candidate probiotic microorganisms: study designs, principal findings and evidential limitations.

Microorganism(s)	Study Design and Product	Evidence Evaluated	Principal Findings	Evidential Interpretation and Limitations	Ref.
*Latilactiibacillus sakei LTH 681 and Lacticaseibacillus paracasei LTH 2579*	Fermented sausages inoculated with both strains; sausages containing *L. paracasei* LTH 2579 were consumed by three volunteers	Survival during sausage manufacture and recovery following gastrointestinal transit	*L. paracasei* LTH 2579 remained viable in the fermented sausage and was recovered from faecal samples following consumption.	Demonstrates compatibility with the product matrix and recovery following gastrointestinal transit; the three-person sample and short-term faecal recovery do not establish stable intestinal colonisation or health benefits	[184]
*Lacticaseibacillus paracasei* LTH 2579	Human dietary intervention involving consumption of fermented sausage containing the strain	Blood-lipid, immunological and faecal-recovery outcomes	Consumption did not significantly alter serum total, HDL or LDL cholesterol or triacylglyceride concentrations. Antibodies against oxidised LDL increased at the end of the intervention. CD4 T-helper lymphocytes increased after two weeks but returned towards baseline by the end of the study; faecal recovery varied among participants.	Provides direct human evidence, but the measured effects were outcome- and time-dependent; the findings should not be generalised to broad cardiovascular or immune system benefits	[185]
*Lactiplantibacillus plantarum* MF1291, MF1298 and DC13; *Lactiplantibacillus pentosus* MF1300; and *Ligilactobacillus salivarius* DC5	Five strains administered to 17 volunteers as freeze-dried cultures; selected strains were also evaluated using fermented sausage as the delivery matrix	Gastrointestinal survival, faecal recovery and short-term persistence	MF1298 and DC13 were recovered most frequently following administration. Recovery and persistence differed among strains, participants and delivery formats, with limited persistence after intake ceased.	Supports strain-dependent gastrointestinal survival and temporary persistence; faecal recovery does not demonstrate permanent colonisation or a host health benefit	[186]
*Enterococcus faecium* CRL183 and *Lactobacillus acidophilus* CRL1014	Low-fat fermented sausages manufactured with the individual strains; product microbiology, physicochemical characteristics and fatty acid composition evaluated	Technological feasibility, strain viability and product-level safety and composition	*E. faecium* CRL183 retained high viable populations, whereas *L. acidophilus* CRL1014 declined relative to the control. Fat-reduced formulations contained more oleic acid and less saturated fatty acids, and the products met the microbiological and physicochemical criteria assessed.	Demonstrates product feasibility and compositional reformulation, not improvement of consumers’ blood-lipid profiles; routine product-safety measurements do not replace comprehensive strain-level safety assessment of *E. faecium* CRL183	[187]

Abbreviations: HDL, high-density lipoprotein; LDL, low-density lipoprotein. Recovery of an administered strain from faecal samples indicates survival through gastrointestinal transit or temporary persistence but does not, by itself, demonstrate stable colonisation or a health benefit. Terminology has been updated where appropriate to reflect current bacterial nomenclature.

Collectively, the studies summarised in Table 4 demonstrate that fermented sausages can support the manufacture and delivery of selected live microorganisms, although the strength and nature of the evidence vary considerably. Product studies confirm that strains can remain viable under defined manufacturing and storage conditions without necessarily compromising the product characteristics assessed, while faecal-recovery studies provide evidence of gastrointestinal transit or transient recovery following consumption [184,186,187]. Klingberg et al. reported that delivery of *Lactiplantibacillus plantarum* MF1298 in fermented sausage increased the number of volunteers from whom the strain was recovered from four to ten, suggesting that the product matrix may have enhanced strain survival during gastrointestinal transit [186]. These outcomes establish technological feasibility and delivery potential, but neither survival within the product nor detection following ingestion demonstrates stable intestinal colonisation or a beneficial effect on the host. Direct evidence of health-related outcomes remains comparatively limited: the intervention involving *Lacticaseibacillus paracasei* LTH 2579 identified selective and time-dependent immunological responses but no significant improvement in the blood-lipid parameters evaluated [185]. The available literature therefore supports fermented sausages as potential carrier matrices for appropriately characterised live strains; however, designation of the resulting products as probiotic requires strain-specific safety assessment, confirmed viable delivery at the studied dose and reproducible evidence of host benefit from adequately designed human investigations.

Caution is required when strains of *Enterococcus faecium* are proposed for incorporation into fermented foods. Although members of this species occur in the gastrointestinal tract and in traditional food fermentations, *E. faecium* also includes opportunistic clinical lineages associated with virulence determinants and acquired antimicrobial resistance. Safety therefore cannot be inferred from species identity, food origin or a history of technological use alone [188]. In the sausage study summarised in Table 4, *E. faecium* CRL183 retained high viable populations, and the finished products satisfied the microbiological and physicochemical criteria evaluated [187]. Long-term administration of CRL183 to rats was also not associated with adverse changes in the body-weight and serum-biochemical parameters examined [189]. These findings provide evidence of product-level feasibility and tolerance under the conditions studied, but they do not constitute a comprehensive strain-level microbiological safety assessment. Before deliberate application as a live food culture, the strain should be evaluated for susceptibility to clinically important antimicrobials; acquired resistance genes and their potential mobility; recognised virulence determinants and relationship to problematic clinical lineages; haemolytic or cytotoxic activity; and the capacity to produce biogenic amines or other undesirable metabolites [188]. Whole-genome analysis should be combined with appropriate phenotypic testing because genomic screening alone cannot establish the absence of all hazardous phenotypes. Consequently, the suitability of CRL183, or any other *E. faecium* strain, must be established for the intended food, consumer population and conditions of use rather than extrapolated from routine finished-product testing or from evidence concerning other strains [190].

### 5.2. Protective Cultures and Bacteriocins

Protective cultures are intentionally introduced viable microorganisms selected principally to inhibit undesirable or pathogenic microorganisms and thereby enhance the microbial safety or stability of a food product [191,192,193]. Unlike conventional starter cultures, whose primary purpose is to direct fermentation and produce predictable technological or sensory characteristics, protective cultures are employed primarily for biopreservation. This distinction is functional rather than absolute, as a single strain may contribute both technological and protective activities when it possesses suitable metabolic and ecological properties. In fermented meat systems, protective cultures may help suppress pathogens or spoilage organisms during manufacture and storage through interactions occurring within the product microbiota, potentially strengthening the control provided by acidification, reduced water activity, curing agents, refrigeration and other established hurdles [41,191,192]. Their effectiveness is nevertheless strain-, target-, matrix- and process-dependent and must be demonstrated under the conditions of the intended application. Protective cultures should therefore be regarded as complementary biological hurdles rather than universal replacements for established preservation measures.

The antagonistic activity of protective cultures generally arises from several interacting ecological and metabolic mechanisms rather than from a single antimicrobial compound. Rapid establishment within the product microbiota may enable an introduced strain to compete with undesirable microorganisms for nutrients and ecological niches, while acidification and the associated accumulation of organic acids can create conditions that restrict the growth of susceptible organisms [41,191,192,193]. Depending on the strain and manufacturing environment, further inhibition may result from the production of hydrogen peroxide, bacteriocins or other antimicrobial metabolites, as well as from broader changes to the microbial community that limit the establishment or persistence of competing populations [191,192,193]. The relative contribution of these mechanisms depends on culture density, metabolite production, target-organism susceptibility and environmental conditions including pH, salt concentration, water activity, temperature and interactions with the resident microbiota. Consequently, inhibitory activity observed in culture media cannot be assumed to persist with equivalent efficacy in fermented meat, where microbial growth and antimicrobial diffusion or activity may be modified by the composition and structure of the food matrix. Protective-culture performance should therefore be validated in the finished product against defined target organisms and under realistic manufacturing, storage and reasonably foreseeable handling conditions.

Among the antimicrobial metabolites produced by protective cultures, bacteriocins have received particular attention as contributors to food biopreservation. Bacteriocins are ribosomally synthesised peptides or proteins that inhibit susceptible bacteria, frequently including organisms closely related to the producer strain, although the breadth of activity varies among compounds [194,195]. Those produced by lactic acid bacteria commonly act against Gram-positive targets through interaction with the cytoplasmic membrane, where pore formation or disruption of essential cellular processes can cause the loss of membrane integrity, cellular inhibition or death; some bacteriocins additionally interfere with cell wall biosynthesis [194,195,196]. Producer strains possess specific immunity mechanisms that protect them from their own bacteriocins. In fermented meat systems, bacteriocinogenic cultures may contribute to the suppression of pathogens such as *Listeria monocytogenes* and selected spoilage organisms, provided that the strain grows and produces an active concentration of the compound under the prevailing processing conditions [196,197,198]. However, bacteriocin production should not be treated as synonymous with protective-culture efficacy. Inhibition may also depend on acidification, competitive establishment and other metabolites, while a bacteriocinogenic strain may perform poorly if expression, release, stability or contact with the target organism is restricted within the product. Bacteriocins should therefore be understood as one strain- and context-dependent mechanism within the broader antagonistic activity of protective cultures.

The application of bacteriocin-based biopreservation in fermented meats may involve inoculation with a bacteriocin-producing protective culture or direct incorporation of a purified, semi-purified or bacteriocin-containing antimicrobial preparation [196,197,198]. Use of the producing organism may permit in situ production of the bacteriocin during fermentation, provided that the strain establishes itself and remains metabolically active under the prevailing conditions. Direct addition offers greater control over the initial antimicrobial concentration but does not provide continued production and may be limited by uneven distribution, degradation or loss of activity within the meat matrix. Product-level studies have demonstrated that both approaches can reduce populations or restrict the growth of susceptible organisms, particularly *Listeria monocytogenes*, although the magnitude and persistence of inhibition vary with the bacteriocin, delivery method, target strain, inoculation level and processing environment [196,197,198]. Their activity may be strengthened through combination with acidification, salt, curing agents, drying, refrigeration or other compatible hurdles; however, an observed reduction should not be interpreted as evidence of pathogen elimination unless this has been specifically demonstrated. Consequently, bacteriocin-based interventions require challenge testing in the intended product to establish the extent and duration of control throughout manufacture, storage and reasonably foreseeable handling conditions.

Interactions with meat proteins and fat can reduce the freely available bacteriocin concentration, while endogenous or microbial proteolytic enzymes may partially degrade susceptible compounds [194,195,196,197,198]. The heterogeneous structure of the product may further restrict diffusion and produce uneven contact between the bacteriocin and target cells, particularly when the antimicrobial is added directly rather than generated near the susceptible population. In bacteriocinogenic protective cultures, efficacy additionally depends on successful establishment of the producer strain and sufficient expression of the active compound under the prevailing conditions of salt concentration, pH, water activity, temperature and nutrient availability. Target organisms may also vary in susceptibility according to strain, physiological state and prior exposure to environmental stresses, and prolonged application may favour the persistence or selection of less-susceptible populations. Consequently, activity demonstrated through agar-diffusion assays, culture media or cell-free preparations cannot be assumed to produce an equivalent reduction in a fermented meat product. Validation should therefore quantify the magnitude and persistence of inhibition within the intended formulation and process while also determining whether the intervention prevents growth, produces a temporary population reduction or achieves a defined level of pathogen control throughout manufacture and storage.

In addition to these matrix- and process-dependent limitations, the practical use of bacteriocins is governed by compound-specific safety evidence and regulatory authorisation. Bacteriocins should not be treated as a uniformly safe or universally permitted category merely because they are ribosomally synthesised, produced by food-associated microorganisms or susceptible to digestive proteases. Assessment must instead consider the identity and structure of the compound, its production organism and manufacturing process, the composition and purity of the resulting preparation, potential toxicity or allergenicity, effects on non-target microbiota, anticipated dietary exposure and the possibility of selecting less-susceptible populations [199,200]. Regulatory acceptance is similarly specific to the bacteriocin or preparation, intended technological function, food category, application route, level of use and jurisdiction. Nisin, for example, is authorised in the European Union as the food additive E234 and has undergone compound-specific safety evaluation; however, its authorisation applies only to the foods and conditions listed in the Union positive list and does not confer equivalent approval on pediocins, sakacins or other experimental bacteriocins [199,200]. Moreover, evidence supporting the use of a purified preparation cannot automatically be extrapolated to application through a viable producer culture, for which the identity, safety and behaviour of the microorganism must also be established. Consequently, technological efficacy, toxicological acceptability and legal authorisation must each be confirmed for the specific bacteriocin-based intervention and intended fermented meat application before commercial use.

## 6. Processing and Packaging Innovations

Emerging processing and packaging technologies offer potential routes to improve microbial control, accelerate manufacturing, support preservative reduction and extend the shelf life of fermented meat products. These approaches include non-thermal physical treatments, process-intensified product formats, functional-culture engineering, active and bio-based packaging materials and intelligent systems for monitoring product condition. However, their technological promise does not necessarily translate into improved sustainability or industrial feasibility. Processing time, energy demand, water consumption, equipment and packaging requirements, product losses and end-of-life impacts must be considered alongside microbial safety, physicochemical stability, sensory quality, regulatory acceptance and production cost. Moreover, much of the available evidence derives from other meat products or laboratory-scale food systems rather than commercial dry-fermented meat manufacture. This section therefore critically evaluates both demonstrated and prospective applications, distinguishing measured outcomes from proposed benefits and identifying the product-specific validation required before these innovations can be implemented at industrial scale.

### 6.1. Green and Minimal Processing Technologies and Functional Cultures

#### 6.1.1. High-Pressure Processing (HPP)

High-pressure processing (HPP) is a non-thermal preservation technology in which packaged foods are exposed to hydrostatic pressures commonly ranging from 100 to 600 MPa. Pressure is transmitted rapidly and approximately uniformly throughout the product, enabling the inactivation of vegetative microbial cells with limited bulk heating [201,202,203]. Its antimicrobial activity arises principally from membrane disruption, protein denaturation and interference with essential cellular processes, although susceptibility varies according to the microorganism, its physiological state, the pressure–time–temperature combination and the food matrix [201,203,204]. Because HPP primarily modifies non-covalent interactions, it may retain certain heat-sensitive constituents and sensory characteristics more effectively than intensive thermal processing [201,202]. Nevertheless, pressure-induced changes in myofibrillar proteins, myoglobin and cellular structure can affect water-holding capacity, texture, colour and lipid oxidation [201,204,205,206]. These responses depend on treatment intensity and temperature, product composition and the stage of manufacture at which HPP is applied.

Direct evidence in dry-fermented meats indicates that HPP can provide an additional microbial hurdle and, under selected conditions, support process intensification. In low-acid fermented sausage, combining HPP with the bacteriocin enterocin AS-48 improved the control of inoculated foodborne pathogens, demonstrating its potential integration with biological preservation [207]. HPP has also been applied during sausage drying to improve microbial safety while shortening processing time [208]. This represents a potentially valuable efficiency gain because drying is prolonged and resource-intensive, although shorter processing does not itself demonstrate reduced energy use or environmental impact. Evidence from emulsified sausage, cooked ham and other ready-to-eat meats further suggests that appropriately selected HPP conditions can maintain acceptable sensory and technological characteristics [209,210,211]. However, these findings establish transfer potential rather than direct validation in dry-fermented products, where reduced water activity, acidity, lipid content and continuing structural development may alter microbial inactivation and quality responses.

Several constraints govern industrial implementation. Bacterial spores are generally more pressure-resistant than vegetative cells, while sublethally injured microorganisms may recover under favourable storage conditions following insufficient treatment [201,203]. Microbial lethality must therefore be validated for the target organism, formulation, processing stage and intended shelf life. Excessive pressure may additionally impair colour, texture, water distribution and oxidative stability, particularly in lipid-rich products [201,204,205,206]. Commercial adoption requires pressure-resistant flexible packaging, batch or semi-continuous operation and substantial capital investment. Although pressure holding requires comparatively little additional energy after the target pressure is reached, overall environmental assessment must account for compression, pumping, decompression, temperature control, throughput and supporting infrastructure [212]. HPP should therefore be regarded as a product-specific supplementary hurdle with increasingly supported contributions to microbial safety and process efficiency, while its quality, economic and environmental outcomes remain dependent on the conditions and scale of the intended fermented meat application.

#### 6.1.2. Cold Plasma and Plasma-Activated Media

Cold plasma is a non-thermal technology in which ionised gas generates reactive oxygen and nitrogen species, charged particles and ultraviolet radiation capable of damaging microbial membranes, proteins and nucleic acids [213,214]. Its operation at or near ambient temperature has prompted interest in meat-surface decontamination with limited thermal exposure [213,215]. In-package treatment may also reduce post-process recontamination by generating reactive species within a sealed package, as demonstrated in ready-to-eat ham [215]. Microbial inactivation nevertheless depends on the plasma-generating system, treatment duration, gas composition, distance from the source, package atmosphere and product characteristics. Consequently, the irregular surfaces, high lipid content, reduced water activity and complex microbiota of dry-fermented meats may produce responses that differ from those of ham, poultry and fresh-meat systems [213,214]. Plasma-activated liquids have additionally been investigated as sources of reactive nitrogen species for curing. Plasma-activated water supported cured-colour formation and microbial control in beef jerky, indicating that plasma-derived nitrite may reproduce selected functions of conventionally added nitrite [216]. Plasma-treated blood-plasma solutions have similarly been evaluated as curing agents in restructured beef jerky [217]. These approaches should not, however, be characterised as inherently “nitrite-free.” Although synthetic nitrite may not be added directly, nitrite and related reactive nitrogen species are generated during plasma activation and contribute to nitric oxide formation and myoglobin nitrosylation. Furthermore, evidence from dried or restructured beef products establishes transfer potential rather than equivalent curing performance, microbial safety or sensory acceptability in fermented sausage.

Several barriers remain to application in fermented meat manufacture. Reactive species may promote lipid and protein oxidation, pigment modification and undesirable flavour development, particularly as treatment intensity increases [213,214]. Surface exposure may also produce non-uniform inactivation within pores, crevices and casing interfaces and cannot be assumed to control microorganisms within the product. Product-specific evaluation must therefore address treatment-derived compounds, microbial recovery following sublethal injury, packaging compatibility and the stability and concentration of reactive species in plasma-activated liquids. Industrial implementation must additionally consider treatment uniformity, throughput, worker safety, equipment requirements and regulatory classification. Environmental benefits cannot be inferred from non-thermal operation alone but require comparison of electricity use, process gases, packaging requirements and treatment efficacy with those of the relevant conventional process. Cold plasma should therefore be regarded as a promising platform for surface decontamination and plasma-assisted curing, although direct evidence of its safety, quality and environmental performance in dry-fermented meats remains limited.

#### 6.1.3. Ultrasound and Pulsed Electric Fields

Ultrasound and pulsed electric fields (PEFs) are non-thermal physical technologies investigated for their effects on mass transfer, cellular structure and biochemical reactions in meat systems. These properties may support process intensification or modify flavour, colour and oxidative stability. However, the technologies operate through different mechanisms and possess distinct evidence bases; accelerated processing or altered product quality does not itself demonstrate improved safety or environmental performance.

High-power ultrasound commonly operates at frequencies of approximately 20–100 kHz, generating acoustic cavitation, microstreaming and localised shear forces that can disrupt tissue structures, enhance mass transfer and influence enzymatic activity [218,219]. Applied after stuffing, ultrasound has promoted proteolysis and modified free-amino-acid and volatile-compound formation in dry-fermented sausage, demonstrating its capacity to influence biochemical development during fermentation and ripening [220]. It has also promoted nitrosylmyoglobin-associated colour formation in nitrite-free pork batter [221]. Although potentially relevant to nitrite reduction, this result does not establish equivalent colour stability or microbial protection in fermented sausage. Ultrasound-assisted pigment formation cannot replace the antimicrobial and antioxidant functions of nitrite without separate validation. Ultrasound may also support ingredient preparation by enhancing the extraction of phenolic and other bioactive compounds from plant materials. In this application, subsequent antioxidant or antimicrobial effects in fermented meat should be attributed to the composition of the resulting extract rather than to direct sonication of the product [222]. Excessive acoustic intensity or treatment duration may additionally cause heating, radical formation and structural disruption, potentially impairing oxidative stability, texture or flavour [218,219].

A PEF applies short electrical pulses to food positioned between electrodes, inducing reversible or irreversible electroporation of biological membranes. Increased cellular permeability may facilitate moisture and solute transport and modify microbial, enzymatic and quality responses [223,224]. Treatment performance depends on electric-field strength, pulse characteristics, specific energy input, electrode configuration and product conductivity, together with matrix structure and degree of comminution [223]. Process-relevant evidence indicates that PEFs may accelerate moisture removal during dry-cured-sausage manufacture, although the effect depends strongly on casing permeability. In pilot-scale Spanish dry-cured *longaniza*, treating minced meat at 1 kV/cm, with a pulse width of 200μs and specific energy input of 28 kJ/kg, reduced the time required to reach the target weight from 17 to 9–10 days in sausages enclosed in gauze, representing a 41–47% reduction [225]. No significant acceleration occurred with natural pork casings, indicating that casing resistance outweighed the increased permeability of the meat matrix. The study therefore demonstrates PEF-assisted drying under defined conditions but not a general effect across casing systems. Moreover, starter-culture performance, microbiological safety and sensory quality require more comprehensive validation before reduced drying time can be interpreted as equivalent manufacture.

Industrial adoption of either technology requires reproducible treatment throughout dense and irregular products. Ultrasound performance depends on acoustic intensity, transducer configuration and coupling conditions, whereas a PEF depends on electrical parameters, electrode design, conductivity and field distribution. For PEFs, these properties may change during fermentation and drying as acidity, salt concentration and moisture content evolve. Validation must therefore examine treatment uniformity, sublethal microbial injury, starter-culture activity, oxidation, texture, colour and sensory acceptance throughout manufacture and storage [226]. Shorter drying or ripening may improve processing efficiency, but environmental advantages require comparative assessment of energy demand, throughput, equipment and product losses. Ultrasound has direct evidence for modifying biochemical development in dry-fermented sausage, while PEF technology has demonstrated casing-dependent drying acceleration in a directly relevant sausage system. Both remain promising process aids requiring broader safety, quality and industrial-scale validation [227,228].

#### 6.1.4. Snack Formats, Alginate Co-Extrusion and Accelerated Drying

Dry-fermented meats are increasingly produced as narrow-calibre sticks, thin bars and sliced products. Beyond providing convenient portioned formats, reducing product diameter or thickness increases the surface-area-to-volume ratio and shortens the distance over which water must migrate, potentially accelerating moisture removal. These advantages are not intrinsic to snack formats: unless temperature, relative humidity and air velocity are adapted to product geometry, rapid drying may cause surface hardening, uneven moisture distribution, excessive acid perception or textural defects. Shorter processing must also provide microbiological control equivalent to that of conventional manufacture through process-specific validation.

Co-extrusion enables the continuous formation of small-calibre products while applying a thin casing or coating around the meat batter. Alginate is suitable for this process because ionic cross-linking with calcium rapidly forms a continuous gel during extrusion. In dry-fermented fuet, co-extruded alginate coatings produced faster drying kinetics than collagen casings, without significant casing-related differences in final water activity, monitored technological and spoilage microorganisms or sensory properties [229]. Alginate-coated sausages nevertheless exhibited lower pH values than collagen-enclosed products. These findings support alginate as a technologically feasible alternative casing system that can facilitate moisture transfer in small-calibre dry-fermented sausage.

Successful casing replacement depends on more than drying rate. Coating composition and thickness, calcium concentration, adhesion, permeability and mechanical resistance can influence product integrity throughout stuffing, fermentation, drying and storage. The coating must accommodate shrinkage while maintaining an acceptable bite and preventing cracking, detachment or localised moisture migration. Its environmental performance also depends on whether shorter processing and chamber occupancy produce measurable reductions in energy use, material consumption, product loss or cost; edibility or bio-based origin alone does not establish an environmental advantage. The proprietary Quick-Dry-Slice (QDS) process provides a mechanistically distinct means of accelerating dehydration. Sausage is first fermented to the required acidification, then frozen, sliced and dried using sequential convective and vacuum treatments [230]. Drying individual slices substantially reduces the moisture-transfer distance relative to intact sausage. QDS therefore differs from narrow snack formats: it modifies product geometry after fermentation rather than maintaining a reduced calibre throughout manufacture.

Comparative studies indicate that QDS can produce dry-fermented sausages broadly comparable to conventionally dried products. Kameník et al. reported similar chemical, instrumental and sensory characteristics between QDS and traditionally manufactured sausages, together with improved microbiological quality following QDS processing [231]. However, composition, fermentation intensity, slice thickness, target moisture content and drying conditions may affect texture, flavour and surface appearance. QDS drying temperature has also influenced lightness, instrumental texture and sensory characteristics, demonstrating that accelerated dehydration must be balanced against retention of the expected product identity [232].

Microbiological validation remains particularly important because accelerated drying changes the duration and intensity of the hurdles encountered by surviving microorganisms. In fermented fish sausage, challenge testing demonstrated that QDS contributed to microbial control, while subsequent HPP provided an additional intervention against inoculated *Listeria monocytogenes* and *Salmonella enterica* [233]. The fish matrix and combined treatment prevent direct extrapolation to conventional pork or beef sausage. Validation must therefore establish pathogen behaviour throughout fermentation, freezing, slicing, drying and storage for each intended formulation. Slicing also increases exposed surface area and handling, potentially introducing opportunities for post-fermentation contamination.

Reduced-calibre formats, alginate co-extrusion and QDS are consequently complementary rather than interchangeable routes to process intensification. Reduced-calibre products shorten the diffusion distance throughout manufacture; co-extrusion combines continuous formation with a thin, permeable coating; and QDS accelerates dehydration by slicing an already fermented product. Each may shorten production, but suitability depends on microbial safety, drying uniformity, structural and sensory quality, equipment compatibility and economic performance. Reduced drying time and chamber occupancy represent valuable efficiency outcomes, although environmental benefits require comparative energy, material-use and life-cycle assessment.

#### 6.1.5. Genome-Informed and CRISPR-Assisted Development of Functional Cultures

Functional starter cultures for fermented meats have traditionally been developed by isolating and phenotypically screening strains for characteristics such as rapid acidification, pathogen inhibition, processing-stress tolerance and contributions to colour, aroma or oxidative stability. Whole-genome sequencing, comparative genomics and metabolic analysis now permit more detailed identification of the genetic determinants associated with these functions, supporting targeted strain selection and safety assessment without necessarily modifying the organism. Genome-informed selection should therefore be distinguished from genetic engineering: detecting genes, predicting metabolic pathways or selecting naturally occurring strains using genomic markers does not constitute genome editing.

CRISPR-Cas systems provide a more targeted means of modifying LAB genomes through gene deletion, insertion, repression or altered expression. Research involving food-associated LAB indicates that these tools can support the investigation of metabolic pathways; improve resistance to acid, salt, oxidative stress or bacteriophage infection; and modify metabolite production relevant to fermentation, flavour, texture and microbial inhibition [234,235]. Dairy fermentation research has further demonstrated the modification of LAB genomes to improve technologically important phenotypes [236]. These developments provide a conceptual basis for engineering fermented meat cultures with enhanced acidification, bacteriocin production, stress tolerance, colour-forming activity or flavour generation. Evidence from dairy cultures and laboratory strains does not, however, establish equivalent performance in meat fermentation, where cultures encounter salt, nitrite, low temperatures, declining pH and water activity, competitive microbiota and a lipid- and protein-rich matrix.

Application in fermented meats is further constrained by the complexity of the desired phenotypes. Acidification, antimicrobial production, nitrate or nitrite metabolism, oxidative protection and aroma development arise from interacting metabolic pathways and may impose competing demands on cellular resources. Enhancing one characteristic could reduce culture viability, disrupt fermentation kinetics or produce unintended metabolic or sensory effects. Edited strains must also remain compatible with co-cultures and resident microbiota without compromising the ecological stability underlying product safety and identity. Furthermore, the detection of antimicrobial-resistance genes within artisanal fermented meat microbiota reinforces the need to screen candidate cultures for acquired and potentially transferable resistance determinants [237]. Precision of editing does not remove the need for genetic-stability assessment, product-level challenge testing, compositional analysis and sensory evaluation.

Regulatory requirements will be a major determinant of implementation. Within the European Union, foods containing or produced using genetically modified microorganisms may fall within the frameworks established by Regulation (EC) No 1829/2003 and Directive 2001/18/EC, although the applicable requirements depend on the nature of the modification and whether viable microorganisms or modified genetic material remain in the product [238,239]. This distinction is particularly relevant to fermented meats because starter cultures may remain viable at consumption. EFSA has concluded that new genomic techniques applied to microorganisms do not necessarily create fundamentally different categories of hazard from established genetic-modification methods and has identifying areas in which existing risk-assessment guidance may require refinement [240]. Assessment of an edited culture would consequently need to address the intended modification, unintended changes, genetic stability, transferability, technological behaviour and potential consequences for human health and the environment. CRISPR should therefore currently be regarded as a research and strain-development platform rather than an established commercial fermented meat technology, with regulatory authorisation and consumer acceptance remaining substantial barriers to implementation.

### 6.2. Edible Films, Smart Sensors and Intelligent Packaging

#### 6.2.1. Edible Coatings, Active Casings and Bio-Based Packaging Materials

Edible films and coatings are thin layers of food-grade material applied to a food surface, whereas active casings are formed or modified to perform functions beyond physically containing the product. Protein- and polysaccharide-based materials, including collagen, alginate, cellulose derivatives, chitosan and whey proteins, can provide partial barriers to moisture, oxygen or solute transfer and act as carriers for antimicrobial or antioxidant compounds [241,242,243]. Their surface location is relevant to ready-to-eat fermented meats because post-processing contamination and aerobic deterioration may originate at the casing or exposed cut surface. However, barrier and delivery functions should be distinguished: an effective antimicrobial carrier may provide inadequate resistance to water vapour or oxygen, while a strong barrier may disrupt the moisture exchange required during fermentation and drying [244].

Direct fermented sausage evidence has been provided using cellulose casings impregnated with chitosan. Tantala et al. demonstrated reduced populations of *Listeria monocytogenes* on ready-to-eat fermented sausage during storage [243]. This represents a directly relevant intervention against post-processing surface contamination, although the material should be described as an antimicrobial-impregnated cellulose casing rather than necessarily as an edible coating because such casings may be removed before consumption. Moreover, the effect arose from chitosan incorporated into a casing rather than replacement of the complete package with an edible film. The findings support a supplementary antimicrobial hurdle but do not demonstrate pathogen elimination or replace hygienic manufacture, validated fermentation and appropriate storage. Evidence from adjacent sausage systems further illustrates the delivery function of active casings. Alirezalu et al. incorporated nisin and ε-polylysine nanoparticles into polyamide–alginate casings for nitrite-free frankfurter-type sausage, while olive leaf, green tea and stinging nettle extracts were added to the formulation [245]. The combined treatments inhibited selected pathogenic and spoilage microorganisms during refrigerated storage, with particularly strong effects in ε-polylysine-containing treatments. However, the respective contributions of casing-bound antimicrobials and formulation-level extracts cannot necessarily be separated. Furthermore, cooked emulsified frankfurters differ from dry-fermented sausages in moisture content, microbial ecology, casing function and storage conditions; the results therefore demonstrate transfer potential rather than direct fermented meat validation.

Performance depends on interactions among the carrier, active compound, food surface and target microorganism. Antimicrobials may bind to proteins or lipids, diffuse too rapidly, remain insufficiently mobile or lose activity as pH, water activity and temperature change. A broad-spectrum agent applied before fermentation could also inhibit desirable starter or ripening organisms. The timing and location of application must consequently reflect the intended function: an active casing present throughout fermentation and drying must maintain microbial compatibility and controlled permeability, whereas a coating applied to sliced finished product can target post-processing contamination. Narrow-spectrum agents or post-ripening application may therefore be preferable where beneficial microbial activity must be preserved [246]. Coatings and casings must retain adhesion, flexibility and integrity as sausages contract during drying. Excessive resistance to moisture transfer may prolong or disrupt drying, whereas excessive permeability may cause rapid surface dehydration and case hardening. Active compounds may additionally affect aroma, flavour, colour and casing bite. Evaluation should therefore encompass microbial challenge testing, release or migration behaviour, barrier and mechanical properties and sensory performance throughout processing and storage [247]. Many polymers investigated for these applications (including cellulose derivatives, starch, chitosan and alginate) are also proposed as alternatives to fossil-derived packaging [248]. Bio-based origin, biodegradability and compostability nevertheless describe distinct properties and should not be treated as synonymous. Inadequate moisture resistance, mechanical strength or sealability may require blending, chemical modification, nanofillers or conventional polymers. Although these modifications can improve barrier, mechanical or active properties, they may alter migration and complicate recycling, biodegradation or composting [249,250]. Direct evidence under vacuum or modified-atmosphere conditions relevant to dry-fermented meats remains limited. Suitability must therefore be established against the oxygen, water-vapour, grease, aroma and mechanical requirements of the intended product. Environmental performance likewise cannot be inferred from edibility, renewable origin or laboratory biodegradability. Some biodegradable materials require industrial composting and may not degrade effectively in landfill, natural environments or existing waste-management systems. Multilayer structures, plasticisers, nanomaterials and active compounds may further affect recyclability, compostability and food-contact safety. Assessment must consequently consider raw-material production, manufacturing energy, package weight, secondary packaging, preservation of product quality, food-waste prevention, collection infrastructure and locally available disposal routes.

#### 6.2.2. Intelligent Indicators and Digital Monitoring

Intelligent packaging complements conventional and active packaging by detecting, recording or communicating information about a food product or its surrounding environment. Unlike active packaging, which releases or absorbs substances to preserve food, intelligent packaging primarily performs a diagnostic or informational function. Principal technologies include freshness indicators that respond to product-derived metabolites, time–temperature indicators (TTIs) that integrate thermal exposure, sensors that generate measurable signals and data carriers such as radio-frequency identification (RFID) and near-field communication (NFC). These functions are not interchangeable: an indicator produces an observable response, a sensor detects or quantifies a variable, and a data carrier identifies, stores or transmits information without necessarily measuring product condition [251,252].

Freshness indicators can detect package-headspace changes associated with microbial or physicochemical deterioration. Zhai et al. developed a silver-nanoparticle-containing gellan gum matrix that changed colour in response to hydrogen sulphide and correlated with deterioration in chicken breast and silver carp [253,254]. Other systems incorporate pH-sensitive pigments, including anthocyanins, into polymeric films or electrospun nanofibers [255]. Their responses to volatile bases produced during protein degradation may correlate with total volatile basic nitrogen, microbial growth or sensory deterioration in fresh meat and seafood [256]. Such indicators enable non-destructive condition monitoring, but their response does not necessarily identify the responsible microorganism or metabolite and cannot demonstrate the absence of pathogens that do not generate the target signal.

Transfer to dry-fermented meats requires product-specific validation. Their low pH, reduced water activity, lipid content, curing ingredients, fermentation metabolites and characteristic microbiota differ substantially from fresh-meat and seafood systems. Volatile acids, amines, sulphur compounds, spices, smoke and mould-ripening metabolites could produce baseline signals or interfere with selectivity. Furthermore, deterioration may involve lipid oxidation, colour loss, aroma changes or package failure rather than the rapid accumulation of volatile bases associated with fresh-meat spoilage. Freshness indicators may consequently be most applicable to sliced, refrigerated or modified-atmosphere-packaged fermented meats, although existing studies demonstrate transfer potential rather than direct validation [244]. Indicator responses would need to be calibrated against product-specific microbiological, physicochemical and sensory acceptance limits.

TTIs integrate the duration and temperature of exposure during distribution and storage. Diffusion-based, enzymatic and photochromic systems generate progressive responses according to time and temperature [257,258,259,260,261], potentially revealing cold-chain deviations not apparent from a single temperature measurement. However, a TTI records the thermal history experienced by the label rather than directly measuring product quality or microbial safety. Its usefulness depends on matching its response kinetics and activation energy to those of the relevant deterioration process. Because fermented meats differ in water activity, pH, packaging atmosphere and refrigeration requirements, the same temperature exposure may have different consequences among products. TTIs may therefore be more informative for refrigerated sliced products and semi-dry sausages than for shelf-stable whole products.

Digital technologies extend monitoring by storing and communicating package- or shipment-level information. RFID can support automated identification and traceability, while NFC enables short-range exchange with compatible readers or smartphones. When coupled with temperature, humidity or gas-sensing elements, these platforms may transmit measurements or exposure histories and support supply-chain auditing, inventory management and remaining-shelf-life modelling [262]. RFID and NFC tags should not themselves be described as freshness sensors unless they incorporate a sensing component. Similarly, environmental loggers record conditions surrounding a package rather than proving the microbiological status of each product. Package position, calibration and local environmental variation may also limit the representativeness of recorded conditions. Implementation depends on selectivity, response speed, stability and reproducibility under realistic storage conditions, as well as signal readability across variations in lighting, package colour and consumer perception. False-positive responses may cause unnecessary food disposal, whereas false negatives could create unwarranted confidence in product safety. Components placed within packages must satisfy food-contact and migration requirements, particularly when metallic nanoparticles, reactive dyes or other sensing compounds are used. Within the European Union, active and intelligent food-contact materials are governed by Commission Regulation (EC) No 450/2009 [263]. Cost, manufacturing compatibility, data integrity, device disposal and consumer understanding must also be balanced against potential improvements in traceability and food-waste prevention. Intelligent systems can reveal changes in products or storage conditions but do not themselves prevent deterioration. Trigger-responsive active packaging seeks to bridge this functional gap by coupling a defined stimulus with the release or activation of a preservative compound.

#### 6.2.3. Prospective Application of Trigger-Responsive Active Packaging

Trigger-responsive active packaging is an emerging extension of controlled-release packaging in which a change within the food or package environment initiates or accelerates the release of an antimicrobial or antioxidant. Unlike conventional active films, which release incorporated agents continuously or through passive diffusion, responsive systems use changes in pH, moisture, temperature, enzyme activity or microbial metabolism to initiate on-demand intervention. Release may occur through stimulus-induced swelling or degradation of a polymer matrix, disruption of electrostatic interactions or cleavage of reversible chemical bonds. Such control could reduce premature depletion and unnecessary release of active compounds when preservation is not required [264,265,266,267]. Reversible Schiff-base linkages between chitosan and aldehyde antimicrobials provide one example: environmental changes can promote bond hydrolysis and release immobilised cinnamaldehyde into the package headspace. Other systems employ pH-sensitive hydrogels, polysaccharide gatekeepers or responsive nanocarriers to control compounds such as carvacrol. These principles have been demonstrated predominantly in fresh meat, seafood and horticultural products, where deterioration may cause pronounced changes in pH, volatile bases, moisture or enzymatic activity. No direct application of a genuinely trigger-responsive release system to dry-fermented meat was identified in the literature examined; its relevance therefore remains prospective rather than experimentally validated. Translation to dry-fermented meats presents a distinctive selectivity problem. Fermentation intentionally changes pH, moisture and microbial activity and could activate a responsive material during manufacture rather than subsequent deterioration. Acidification by starter cultures could initiate a pH-responsive system, while declining water activity could alter moisture-triggered release. Fermentation-derived acids and volatiles, proteolytic products, smoke constituents and surface-mould activity could similarly interfere with trigger specificity. Conversely, deterioration of a stable, low-water-activity product may produce changes too limited to activate systems designed for rapidly spoiling fresh foods. Responsive materials must therefore distinguish desirable fermentation and ripening from genuine loss of product stability. Post-ripening packaging of sliced fermented meats represents the most plausible initial application because oxygen ingress, package leakage, surface contamination and refrigerated storage provide more clearly defined preservation targets than fermentation itself. Candidate systems would require calibration against product-specific microbial, oxidative and sensory endpoints, together with evaluation of release kinetics, effective concentrations, migration, packaging-atmosphere compatibility and sensory effects. Safety assessment must encompass the carrier, active substance and any reaction or degradation products generated during activation. Until such validation is available, trigger-responsive packaging should be regarded as a prospective supplementary hurdle rather than an established fermented meat technology.

### 6.3. Critical Comparison and Implementation Considerations

Emerging biological, processing and packaging technologies provide complementary routes to improving the safety, quality and sustainability of fermented meats, but they differ substantially in function, evidential maturity and proximity to implementation. Functional cultures and selected active casings possess comparatively strong direct product-level evidence, whereas high-pressure processing (HPP), cold plasma, ultrasound, pulsed electric fields (PEFs) and accelerated drying exhibit application-dependent levels of validation. Intelligent packaging principally supports post-manufacture monitoring, while genome editing and trigger-responsive packaging remain largely prospective. These technologies should therefore be evaluated according to their intended point of application and supporting evidence rather than presented collectively as equivalent alternatives to conventional preservation.

At culture-development and formulation stages, naturally selected functional cultures and bacteriocin-producing microorganisms are among the most immediately applicable biological interventions. Their integration into established manufacturing practices may support fermentation control, pathogen inhibition, curing, flavour development and oxidative stability. Performance nevertheless depends on strain-level characteristics and interactions with salt, nitrite, pH, temperature, water activity, bacteriophages and competing microbiota. Activity demonstrated in vitro does not ensure survival, metabolite production or uniform functionality within the meat matrix. Genome-informed selection can strengthen culture characterisation without modifying the organism, whereas CRISPR-assisted editing offers more targeted but less mature possibilities for altering stress tolerance, antimicrobial production or metabolic activity. Edited cultures require assessment of genetic stability, unintended effects, ecological compatibility and regulatory status, while consumer acceptance may be constrained by perceptions of risk, naturalness and trust [268]. CRISPR therefore remains primarily a research and strain-development platform rather than an established fermented meat intervention.

During manufacture, processing technologies act through different mechanisms and cannot be treated as interchangeable. HPP has comparatively substantial evidence as a supplementary microbial hurdle, while cold plasma offers more surface-directed decontamination and plasma-assisted curing functions. Ultrasound may modify proteolysis and volatile development, whereas PEFs and reduced calibre or sliced formats can intensify moisture transfer. Alginate co-extrusion and QDS further illustrate how casing permeability and product geometry can govern drying efficiency. In each case, treatment timing is critical: interventions applied before or during fermentation may disrupt starter or ripening organisms, while post-ripening application may permit more selective microbial control. Accelerated drying must also retain equivalent safety and product quality, and shorter processing cannot itself demonstrate reduced energy demand or environmental impact. Edible coatings and active casings provide targeted protection at product surfaces but must be designed for their intended processing stage. Materials present during fermentation and drying require controlled permeability, structural integrity and compatibility with beneficial microbiota; excessive barrier performance may restrict moisture loss or cause uneven drying, while broad-spectrum antimicrobials may inhibit desirable cultures. By contrast, coatings applied after ripening to sliced products may be optimised principally for microbial, oxidative and moisture protection. Their functionality depends jointly on the carrier, active compound, release behaviour and food matrix, and bio-based origin or edibility does not itself establish technical or environmental superiority.

Intelligent indicators and digital monitoring systems are most applicable after ripening, particularly during refrigerated distribution of sliced products. Freshness indicators may detect package-headspace changes, while TTIs, RFID and NFC-enabled systems can support cold-chain verification, traceability and inventory management. These technologies do not themselves preserve food, and their signals cannot be assumed to indicate microbiological safety. Fermentation-derived metabolites, smoke, spices, surface microbiota and product-specific deterioration pathways may interfere with indicators developed for fresh meat or seafood. Their near-term value may therefore lie in identifying temperature or package-integrity failures and improving supply-chain decisions, subject to calibration against defined product-specific acceptance limits. Trigger-responsive active packaging seeks to connect detection with preservation by releasing or activating an antimicrobial or antioxidant following a defined environmental change. Its application to dry-fermented meats remains prospective because changes in pH, moisture and microbial metabolism that indicate deterioration in fresh foods are intentional features of fermentation and ripening. Conversely, deterioration in stable, low-water-activity products may generate an insufficient activation signal. Post-ripening sliced products provide the most plausible initial application because oxygen ingress, package leakage and surface contamination offer more clearly defined targets. Direct validation is required before these systems can be considered established fermented meat preservation strategies.

The integration of multiple interventions may provide broader control than any technology alone, but combined effects should not be presumed additive or synergistic. Fermented meat is governed by interacting processes of microbial growth, acidification, moisture loss, curing, proteolysis, lipolysis and oxidation. An antimicrobial coating could inhibit a functional surface culture; plasma treatment could increase oxidative susceptibility; reduced salt or nitrite could modify microbial ecology and active-agent efficacy; and a restrictive packaging material could alter drying and sensory development. Combination strategies must therefore be validated as complete hurdle systems using controls that distinguish individual contributions and identify antagonistic as well as beneficial interactions. Commercial translation requires evidence extending beyond laboratory antimicrobial efficacy. Technologies must demonstrate reproducibility across independent manufacturing batches, compatibility with relevant equipment and processing speeds, scalability, storage stability, sensory acceptability and economic feasibility. Validation should use representative formulations, whole or sliced formats, realistic packaging atmospheres and storage conditions and relevant spoilage or pathogenic microorganisms. Regulatory assessment must reflect each technology’s mode of action. For example, active and intelligent food-contact materials require evaluation of authorised substances, migration, labelling and consumer information under Commission Regulation (EC) No 450/2009, while novel cultures, engineered microorganisms, nanomaterials, plasma-derived compounds and responsive release products present distinct assessment requirements [263].

Environmental benefits must similarly be demonstrated rather than inferred from descriptors such as bio-based, biodegradable, minimal processing or clean label. Renewable feedstocks or shortened processing may reduce selected burdens, but material extraction, chemical modification, energy demand, equipment, multilayer construction and inadequate disposal infrastructure may offset these gains. Conversely, packaging with greater production impacts may reduce the overall burden if it prevents waste of a resource-intensive meat product. Life-cycle assessment should therefore encompass raw materials, processing energy, package manufacture and transport, product preservation, food-waste prevention and locally available disposal pathways in accordance with ISO 14040 and ISO 14044 [269,270]. Packaging assessments do not always adequately account for packaging-related changes in food waste, despite their potential importance to the overall outcome [271]. Practical adoption will ultimately depend on whether measurable safety, quality or environmental benefits justify the associated technological, regulatory and economic demands. Functional cultures and appropriately designed active casings appear closest to broader implementation because they fit familiar manufacturing operations and possess direct fermented meat evidence. HPP, other non-thermal treatments, accelerated-drying approaches and intelligent monitoring may provide valuable supplementary functions but require application-specific optimisation and scale-up. Genome editing and trigger-responsive packaging remain longer-term research directions because direct fermented meat validation is lim ited and regulatory or consumer barriers remain unresolved. Future research should prioritise comparative pilot-scale studies, complete hurdle-system validation, migration and safety assessment, life-cycle analysis and consumer research. These innovations should supplement, not replace, raw-material control, validated fermentation, starter-culture management, manufacturing hygiene, packaging integrity and shelf-life validation. Examples of these technologies and packaging innovations and their potential or emerging applicability to dry fermented meats, are presented in Table 5.

**Table 5:** Critical comparison of emerging biological, processing and packaging technologies relevant to fermented meat manufacture.

Technology *	Principal Function	Most Suitable Application Stage or Format	Evidence in Fermented Meats	Potential Benefit	Principal Implementation Constraint	Current Position	Refs.
Functional starter cultures and microbial ingredients	Fermentation control, microbial inhibition and development of curing, flavour and quality characteristics	Formulation and fermentation	Comparatively strong direct product-level evidence	Predictable fermentation, biological preservation and potential support for salt or nitrite reduction	Strain–matrix interactions, bacteriophage susceptibility, ecological compatibility and process consistency	Among the closest to broader implementation	[191,194,197,207,272]
Genome-edited cultures, including CRISPR-based approaches	Targeted modification of stress tolerance, antimicrobial production and metabolic activity	Culture development, followed by potential use during fermentation	Predominantly conceptual or laboratory-based; limited direct fermented meat validation	Precise optimisation of safety- and quality-related culture functions	Regulation, genetic stability, unintended effects, ecological compatibility and consumer acceptance	Longer-term research prospect	[234,235,236]
High-pressure processing	Microbial inactivation with limited bulk heating	Principally post-fermentation or post-ripening; whole or packaged ready-to-eat products	Direct product-level evidence, including dry-fermented sausages	Pathogen control and possible shelf-life extension or process intensification	Microbial pressure resistance; texture, colour and oxidation effects; packaging requirements and capital cost	Comparatively mature, but product- and process-dependent	[14,201,202,204,207,208,212,273]
Cold plasma and plasma-activated media	Surface microbial inactivation and generation of reactive curing species	Principally post-ripening surfaces or packaged sliced products; plasma-activated curing ingredients remain transferable applications	Limited fermented meat evidence; broader evidence from adjacent meat systems	Surface decontamination and potential support for alternative curing strategies	Limited penetration, non-uniform exposure, oxidation, sensory effects and compatibility with desirable microbiota	Promising, but requires direct product-level optimisation and scale-up	[212,213,214,215,216,217]
Ultrasound and pulsed electric fields	Modification of tissue structure, biochemical reactions and mass transfer	During formulation, fermentation or drying, depending on the intended function	Selected direct or process-relevant sausage evidence; broader validation remains limited	Modification of proteolysis and volatile development or acceleration of moisture transfer	Treatment uniformity, oxidation, casing dependence, starter-culture effects and scale-up	Promising process aids requiring application-specific validation	[218,219,220,221,222,223,224,225,274]
Reduced-calibre formats, alginate co-extrusion and QDS	Process intensification through reduced moisture-transfer distance or permeable coating systems	Throughout manufacture for reduced-calibre and co-extruded products; post-fermentation for QDS	Direct evidence in selected dry-fermented sausage systems	Shorter drying, continuous formation and convenient portioned formats	Drying uniformity, casing or coating performance, microbial equivalence, sensory identity and equipment compatibility	Technologically feasible but format- and process-dependent	[229,230,231,232,233]
Edible coatings and active casings	Surface protection, moisture regulation and delivery of antimicrobial or antioxidant compounds	Fermentation, drying or post-ripening, depending on permeability and intended function	Direct evidence in selected fermented meat systems	Control of surface contamination, oxidation and moisture transfer	Drying compatibility, release behaviour, migration, sensory effects and inhibition of desirable microbiota	Near-term and application-dependent	[241,242,243,245,248,275,276]
Bio-based and potentially biodegradable packaging materials	Physical, oxygen, moisture or active packaging functions using wholly or partly biologically derived materials	Principally post-ripening packaging of whole or sliced products	Limited direct evidence under conditions relevant to dry-fermented meats	Potential reduction in fossil-resource dependence and, under defined conditions, improved end-of-life options	Barrier and mechanical performance, migration, modification or blending requirements, disposal infrastructure and unverified life-cycle benefit	Application-dependent; environmental advantage requires demonstration	[248,249,250,277,278,279,280]
Intelligent indicators and digital monitoring	Detection or communication of product condition, package environment or storage history	Packaging, distribution and retail, particularly for refrigerated sliced products	Limited direct validation in dry-fermented meats; evidence principally transferred from fresh meat and seafood	Cold-chain verification, traceability, condition monitoring and inventory management	Product-specific calibration, signal interference, false responses, cost and interpretation	Plausible near-term monitoring tool, subject to validation	[251,252,253,254,256,257,258,259,260,262,281]
Trigger-responsive active packaging	Stimulus-initiated or accelerated release of antimicrobial or antioxidant compounds	Most plausibly post-ripening sliced products	No direct dry-fermented meat validation identified in the literature examined	On-demand preservation with reduced premature release or depletion	Distinguishing fermentation from deterioration; release control, migration, sensory compatibility and safety	Prospective technology	[264,265,266,267]

* Technologies are compared according to their principal function, most suitable point of application, maturity of fermented meat evidence, potential benefits, principal implementation constraints and current proximity to practical adoption. The classifications reflect the evidence evaluated in this review and do not imply regulatory authorisation or commercial availability across jurisdictions.

## 7. Conclusions

This review critically examined developments intended to support the production of safer, healthier and potentially more sustainable fermented meat products. Strategies for reducing salt, nitrate, nitrite, smoke-derived contaminants and animal fat demonstrate that reformulation is possible, but the technological functions of these components cannot be considered independently of microbial safety, fermentation performance, oxidative stability, colour, texture, flavour and consumer acceptance. Functional starter cultures, protective microorganisms and bacteriocins may help compensate for selected preservative reductions, although their performance remains dependent on strain characteristics, product composition and processing conditions.

Biological, non-thermal, accelerated processing and packaging technologies offer complementary rather than interchangeable functions. Functional cultures and selected active casings currently possess comparatively direct fermented meat evidence, whereas several emerging processing, intelligent-packaging, genome-editing and trigger-responsive systems require further product-level validation. Effective implementation will depend on reproducibility across manufacturing batches, compatibility with existing processes, scale-up, regulatory compliance, economic feasibility and consumer acceptance. Combinations of interventions should be assessed as complete hurdle systems because their effects cannot be presumed to be additive or synergistic. Fermentation and associated preservation strategies may provide environmental advantages where they reduce processing, storage or product-loss burdens. However, shorter processing times, reduced refrigeration requirements, bio-based materials or extended shelf life do not independently demonstrate lower overall environmental impacts or actual food-waste prevention. Such benefits must be established through comparative assessment of energy and material inputs, processing and distribution requirements, packaging, product losses and relevant end-of-life pathways. Future research should therefore integrate microbiological, physicochemical and sensory validation with pilot-scale manufacturing, life-cycle assessment and consumer research. This evidence-based approach will be necessary to determine whether emerging innovations can deliver measurable improvements in safety, health-related formulation and environmental performance while retaining the defining quality and stability of fermented meat products.

## Figures and Tables

**Table 1:** Selected case studies illustrating mechanistically distinct strategies for reducing or eliminating directly added nitrate and nitrite in fermented meat products.

Strategy *	Hypothesis	Implications	Limitations	Case Study
Direct nitrate/nitrite reduction	French dry-fermented sausages containing nitrate alone or nitrate/nitrite combinations; nitrite tested at 120 and 80 mg/kg	Both 120 and 80 mg/kg nitrite produced the greatest reductions in *Salmonella typhimurium* and *Listeria monocytogenes*; a 47% nitrite reduction provided a sanitary effect comparable to the regulatory dose	Hazard-specific challenge study; does not demonstrate equivalent control of *C. botulinum*, oxidation, colour, flavour or shelf life	[37]
Direct nitrate/nitrite reduction	Dry-fermented sausage with full, 50%-reduced or absent nitrate/nitrite	Reducing curing agents by 50% to approximately 75 mg/kg retained an inhibitory effect against *S. typhimurium* comparable to the full treatment; absence of curing agents resulted in final counts approximately 2.0–2.5 log_10_ CFU/g higher	Evaluated *Salmonella* survival rather than comprehensive product equivalence	[18]
Indirect plant-derived curing	0.5% or 1.0% radish powder; 0.5% or 1.0% beetroot powder; *Staphylococcus carnosus* starter; compared with 150 mg/kg nitrite + 150 mg/kg nitrate and an uncured control	Radish contained approximately 16,263 mg nitrate/kg and beetroot approximately 14,037 mg/kg; vegetable powders generated nitrite during ripening and affected colour, oxidation, moisture, (*a_w_*) and weight loss	Plant ingredients did not behave interchangeably; equivalent antibotulinal protection was not established	[44]
Functional compensation	*Kitaibelia vitifolia* extract used in nitrite-free dry-fermented sausage	Antioxidant activity, phenolic/flavonoid content, instrumental colour, sensory quality and antimicrobial activity were assessed; extract-containing products exhibited antioxidant and organism-specific antimicrobial effects	Antimicrobial findings were largely based on extracts from the sausage tested against selected organisms, not an in-product pathogen challenge	[46]
Functional compensation	Olive leaf extract: 1000 mg/kg with 0 or 75 + 75 mg/kg nitrite/nitrate; 500 mg/kg with 35 + 35 mg/kg; appropriate controls	The 500 mg/kg OLE + 35/35 mg/kg treatment reduced lipid and protein oxidation relative to 75/75 mg/kg curing salts alone; OLE also reduced *N*-nitrosamines	OLE decreased redness and introduced slight bitterness; microbiological quality was monitored, but curing-safety equivalence was not demonstrated	[51]
ZnPP formation	Nitrite-free dry-fermented sausage manufactured under different pH, meat-source and maturation conditions	ZnPP formed only above approximately pH 4.9 and increased markedly during prolonged production, continuing up to day 177; conventional rapid acidification was therefore disadvantageous	ZnPP formation was slow and did not necessarily produce sufficient or uniform conventional redness	[54]
ZnPP formation	Five ZnPP-forming LAB at approximately 6 log_10_ CFU/g; nitrite-added and uninoculated controls; 28-day manufacture	After 28 days, *Latilactobacillus curvatus* and *Lactococcus lactis* subsp. *cremoris* produced the greatest final ZnPP contents, reaching 6.101 ± 0.159 and 6.076 ± 0.120 nmol/g dry matter, respectively, compared with 2.029 ± 0.027 nmol/g in the uninoculated control and 0.573 ± 0.012 nmol/g in the nitrite-added control; LAB inoculation also increased ZnPP-associated fluorescence and product redness	Only two experimental replicates were reported; colour compensation was investigated without demonstrating equivalent pathogen control, oxidative stability or shelf life	[55]
bNOS-mediated nitrosylation	*Staphylococcus vitulinus* (now *Mammaliicoccus vitulinus*) with experimentally enhanced NOS expression under oxidative stress	Enhanced NOS expression increased nitric oxide production, nitrosylmyoglobin formation and fermented sausage redness	Genetically/experimentally enhanced expression demonstrates mechanism more than immediate industrial applicability	[69]
bNOS-mediated nitrosylation	Fermented sausages inoculated with or without *M. vitulinus*, with assessment of bacterial NOS-associated pigment formation	*M. vitulinus* inoculation enhanced redness and nitrosylmyoglobin formation, providing product-level support for the bNOS pathway	Evidence principally concerns colour; preservation, nitrosative chemistry, oxidation and pathogen control require separate validation	[70]
Deliberately uncured reformulation	Nitrate/nitrite-free cold-ripened salami containing 0.2% ascorbic acid or 0.3% lactic acid; conventional control contained 80 mg/kg nitrate/nitrite	All formulations reached (*a_w_* = 0.92) by day 22 and (<0.895) by day 35; ordinary RH treatments produced oxidation profiles broadly comparable to the control	Low RH of 65–80% caused case hardening and increased MDA and other lipid oxidation markers; no inoculated challenge test	[39]
Deliberately uncured reformulation	No nitrate/nitrite; high bioprotective starter inoculum of 7 log_10_ CFU/g; 0.2% or 0.4% glucose; fermentation/ripening at 10–15 °C	Final *a_w_* was 0.908–0.914; weight loss approximately 38%; LAB 8.1–8.3 log_10_ CFUg/; CNS 6.8–7.1 log_10_ CFU/g; no growth of challenged *Listeria innocua*, *Salmonella enterica* or *C. botulinum* occurred; TBARS was 0.8 versus 1.1 mgMDA/kg with 0.2% versus 0.4% glucose	The study showed no growth rather than necessarily demonstrating a required pathogen lethality; the uncured treatments had lower redness and higher TBARS than the cured control	[73]

* Strategies are classified according to their demonstrated technological mechanism rather than the natural or synthetic origin of the intervention. Reported effects on individual curing functions should not be interpreted as evidence of complete curing equivalence unless microbiological safety, oxidative stability, colour, sensory quality and shelf-life performance were collectively evaluated.

**Table 2:** Selected strategies for reducing or partially replacing NaCl in fermented meat products and their principal technological, microbiological and sensory effects.

Product	NaCl-Reduction Strategy	Principal Findings	Technological Implication	Ref.
Fermented cooked sausage	Replacement of 50% of NaCl with KCl, with lysine (0.313%) or a mixture of taurine (750 mg/kg), disodium inosinate (300 mg/kg) and disodium guanylate (300 mg/kg) added to compensate for sensory defects	Replacement with KCl alone adversely affected sensory quality. Lysine and the taurine-ribonucleotide mixture reduced the sensory defects associated with KCl addition.	Flavour-enhancing amino acids and ribonucleotides can improve the sensory feasibility of substantial NaCl replacement with KCl.	[110]
Harbin dry sausage	Control containing 100% NaCl; 70% NaCl and 30% KCl; or 70% NaCl, 20% KCl and 10% flavour-enhancing mixture comprising lysine, maltodextrin, alanine, calcium lactate and citric acid	Partial substitution altered lipid and protein oxidation and volatile-compound development. The composite substitute produced lower lipid and protein oxidation than KCl substitution alone and generated a flavour profile more similar to that of the NaCl control.	Combining KCl with saltiness and flavour enhancers may provide better oxidative and flavour outcomes than substitution with KCl alone.	[87]
Fermented dry sausage	Direct reduction from 2.5% NaCl to 2.0%, 1.5% or 1.0%	Decreasing NaCl increased moisture, water activity and LAB counts and reduced pH. Salt reduction also altered volatile-compound formation and sensory characteristics. The 2.0% NaCl treatment provided the most favourable overall balance among the reduced-salt formulations.	Moderate direct reduction may be feasible, whereas larger reductions can substantially alter fermentation, moisture relations and flavour development.	[83]
Slow-fermented sausage	Control containing 2.7% NaCl; reduced-salt treatment containing 2.26% NaCl; or partial replacement treatment containing 2.26% NaCl and 0.43% KCl	Salt reduction and KCl addition modified the generation of aroma-active compounds. Overall consumer acceptability was maintained, but aroma acceptance was adversely affected in the KCl-containing product.	Relatively limited NaCl reduction or replacement may preserve general acceptance while still changing the product’s characteristic aroma.	[85]
Brazilian dry-fermented sausage	Control containing 2.5% NaCl; 50% direct NaCl reduction; or replacement of 50% of NaCl with KCl, CaCl_2_ or an equal blend of KCl and CaCl_2_	Direct reduction and KCl replacement decreased firmness. Treatments containing CaCl_2_ altered proteolysis and free-amino-acid composition and increased hardness. Related sensory and volatile analyses associated CaCl_2_-containing treatments with greater bitterness and rancid character and with increased formation of lipid-oxidation-derived volatile compounds.	KCl and CaCl_2_ cannot be regarded as technologically interchangeable: KCl primarily affected firmness, whereas CaCl_2_ exerted stronger effects on proteolysis, texture, volatile formation and sensory quality.	[86,108,109]
Low-salt dry-fermented sausage with partial fat replacement	Partial replacement of animal fat with soy flakes combined with the use of the commercial salt blends Aranca Salt™ or Novosal Salt™	Salt blend and fat source jointly affected composition, oxidation indices and LAB counts. Soy addition generally reduced protein and peroxide values but increased LAB counts and malondialdehyde concentrations; responses differed between salt systems.	Because fat and salt were reformulated simultaneously, the independent effect of NaCl replacement cannot be isolated. The study is best interpreted as evidence for combined product reformulation.	[111]
Cacciatore salami	Replacement of 50% of the formulation NaCl with a mixture of KCl, CaCl_2_ and MgCl_2_	Sodium content was reduced by approximately 40%, with limited detrimental effects on sensory attributes. No significant effects were observed on pH, water activity, proximate composition or free-fatty-acid composition, although lipid oxidation increased significantly.	Mixed chloride-salt replacement can substantially lower sodium while maintaining several quality attributes, but oxidative stability requires specific consideration.	[106]

## Data Availability

No new data were created or analyzed in this study. Data sharing is not applicable to this article.
